# Immunoreceptor CD300a regulates ischemic tissue damage and adverse remodeling in the mouse heart and kidney

**DOI:** 10.1172/JCI184984

**Published:** 2025-07-24

**Authors:** Nanako Nishiyama, Hitoshi Koizumi, Chigusa Nakahashi-Oda, Satoshi Fujiyama, Xuewei Ng, Hanbin Lee, Fumie Abe, Jinao Li, Yan Xu, Takehito Sugasawa, Kazuko Tajiri, Taketaro Sadahiro, Masaki Ieda, Keiji Tabuchi, Kazuko Shibuya, Akira Shibuya

**Affiliations:** 1 Department of Immunology, Institute of Medicine,; 2 Doctoral Program of Biomedical Sciences, Graduate School of Comprehensive Human Sciences,; 3 Department of Otolaryngology, Head and Neck Surgery, Institute of Medicine,; 4 R&D Center for Innovative Drug Discovery,; 5 Master’s Program in Medical Sciences, Graduate School of Comprehensive Human Sciences, and; 6 PhD Program in Human Biology, School of Integrative and Global Majors, University of Tsukuba, Tsukuba, Ibaraki, Japan.; 7 TNAX Biopharma Corp., Tsukuba, Ibaraki, Japan.; 8 Department of Sports Medicine Analysis, Institute of Medicine, University of Tsukuba, Tsukuba, Ibaraki, Japan.; 9 Department of Cardiology, National Cancer Center Hospital East, Kashiwa, Chiba, Japan.; 10 Department of Cardiology, Keio University School of Medicine, Shinjuku, Tokyo, Japan.; 11 Life Science Center for Survival Dynamics, Tsukuba Advanced Research Alliance, University of Tsukuba, Tsukuba, Ibaraki, Japan.

**Keywords:** Immunology, Inflammation, Fibrosis, Macrophages, Neutrophils

## Abstract

Acute ischemic organ diseases such as acute myocardial infarction and acute kidney injury often result in irreversible tissue damage and progress to chronic heart failure (CHF) and chronic kidney disease (CKD), respectively. However, the molecular mechanisms underlying the development of CHF and CKD remain incompletely understood. Here, we show that mice deficient in CD300a, an inhibitory immunoreceptor expressed on myeloid cells, showed enhanced efferocytosis by tissue-resident macrophages and decreased damage-associated molecular patterns and pathogenic SiglecF^hi^ neutrophils, resulting in milder inflammation-associated tissue injury than in wild-type mice after ischemia and reperfusion (IR). Notably, we uncovered that CD300a deficiency on SiglecF^lo^ neutrophils increased the signal transducer and activator of transcription 3–mediated production of pro-angiogenic and antifibrotic factors, resulting in milder adverse remodeling after IR. Our results demonstrated that CD300a plays an important role in the pathogenesis of ischemic tissue injury and adverse remodeling in the heart and kidney.

## Abstract 2

CD300a blockade promotes efferocytosis and the SiglecF^lo^ neutrophils activation, which protects against tissue fibrosis and adverse remodelling after IR injury in the heart and kidney.

## Graphical abstract

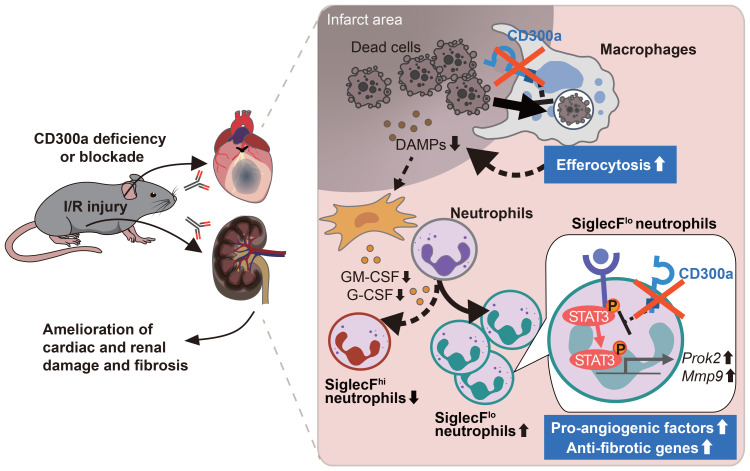

## Introduction

Acute myocardial infarction (AMI) is caused by the occlusion of a coronary artery by plaque or thrombus. Percutaneous coronary intervention and coronary artery bypass grafting are widely used to treat AMI and have improved survival rates; however, the number of patients progressing to chronic heart failure (CHF) is rapidly increasing worldwide (1). Acute kidney injury (AKI) is also caused by ischemia and reperfusion (IR) due to therapy or sporadic arterial occlusion; this results in either reversible or irreversible tissue damage (2). AKI is a complication of various diseases, such as heart failure (called cardiorenal syndrome; refs. 3, 4), liver failure, and sepsis in patients admitted to the hospital, where the prevalence of AKI in patients in the intensive care unit sometimes exceeds 50% (5). AKI often progresses to chronic kidney disease (CKD), which is defined as renal dysfunction that has persisted for more than 3 months (5).

Within the first few hours of cardiac or renal IR, a large number of cardiomyocytes and tubular epithelial cells die and release damage-associated molecular patterns (DAMPs) such as high-mobility group box 1 (*HMGB-1*), interleukin-1α (IL-1α), nucleic acids, and adenosine triphosphate (ATP) (6), which induce inflammation by activating tissue-resident myeloid cells and recruiting inflammatory myeloid cells such as neutrophils and inflammatory monocytes (7). Although the inflammatory responses that accompany angiogenesis and fibrosis are necessary for proper tissue repair, persistent excessive inflammation caused by the subsequent reperfusion exacerbates tissue damage and fibrosis, leading to tissue scarring in the infarcted area, followed by the development of CHF or CKD (5, 8). However, the molecular mechanisms underlying the development of CHF and CKD remain incompletely understood. Moreover, no effective therapy has yet been established to treat cardiac and renal IR-induced injury (IRI) and prevent progression to CHF and CKD, respectively.

Recent studies have reported a unique neutrophil subset highly expressing the cell surface protein sialic acid–binding immunoglobulin-type lectin F (SiglecF, homolog of human Siglec-8) in the mouse heart after AMI (9, 10). Furthermore, a single-cell transcriptome analysis of murine heart tissue 3 days after AMI has demonstrated the existence of two distinct subsets of neutrophils expressing high or low amounts of SiglecF (i.e., SiglecF^hi^ and SiglecF^lo^) (10). SiglecF^hi^ neutrophils have also been identified in injured murine lung and kidney tissue; they produce proinflammatory and profibrotic cytokines and participate in aberrant tissue remodeling (11, 12). In contrast, however, the functional role of SiglecF^lo^ neutrophils in the pathology of ischemic organ diseases remains unclear, although it has been postulated that their function is similar to that of circulating neutrophils (10).

CD300-family molecules are encoded by 8 genes in humans and 12 genes in mice (13) and preferentially expressed on myeloid cells, either activating or inhibiting innate immune responses. One of the family members — CD300a in mice and CD300A in humans — contains the immunoreceptor tyrosine-based inhibitory motif (ITIM) in the cytoplasmic region (14) and mediates an inhibitory signal (15). Phosphatidylserine (PS) exposed on the plasma membrane of dead cells is a functional ligand for CD300a (16). Mice deficient in CD300a on mast cells show prolonged survival in the cecal ligation and puncture sepsis model via augmented secretion of mast cell–derived chemoattractant and neutrophil recruitment (17). In addition, mice deficient in CD300a on CX3CR1^+^CD103^–^CD11b^+^ dendritic cells have upregulated IFN-β–dependent regulatory T cell (Treg) expansion in the barrier tissues such as the intestine, skin, and airway (18). On the other hand, CD300a on macrophages suppresses CD300b-mediated efferocytosis by inhibiting activation of the CD300b-associated adaptor DNAX activation protein 12 (DAP12) (19). CD300a deficiency enhances efferocytosis by inflammatory monocytes infiltrating into the brain after middle cerebral artery occlusion and reperfusion in mice; this results in amelioration of the neurological deficit (19). These results raise the question of whether CD300a blockade can ameliorate ischemic damage in organs other than the brain via enhanced efferocytosis.

Here, we showed that CD300a deficiency reduced inflammation-associated tissue damage and dysfunction in the heart and kidney after IR by increasing the efferocytosis by tissue-resident macrophages and decreasing the release of DAMPs from dead cells. Moreover, we unveiled the important role of SiglecF^lo^ neutrophils in remodeling after IRI. CD300a deficiency on SiglecF^lo^ neutrophils increased signal transducer and activator of transcription 3 (STAT3) phosphorylation and increased the production of pro-angiogenic and antifibrotic factors after IR.

## Results

### CD300a deficiency reduces cardiac IRI and adverse remodeling.

**Figure 1:**
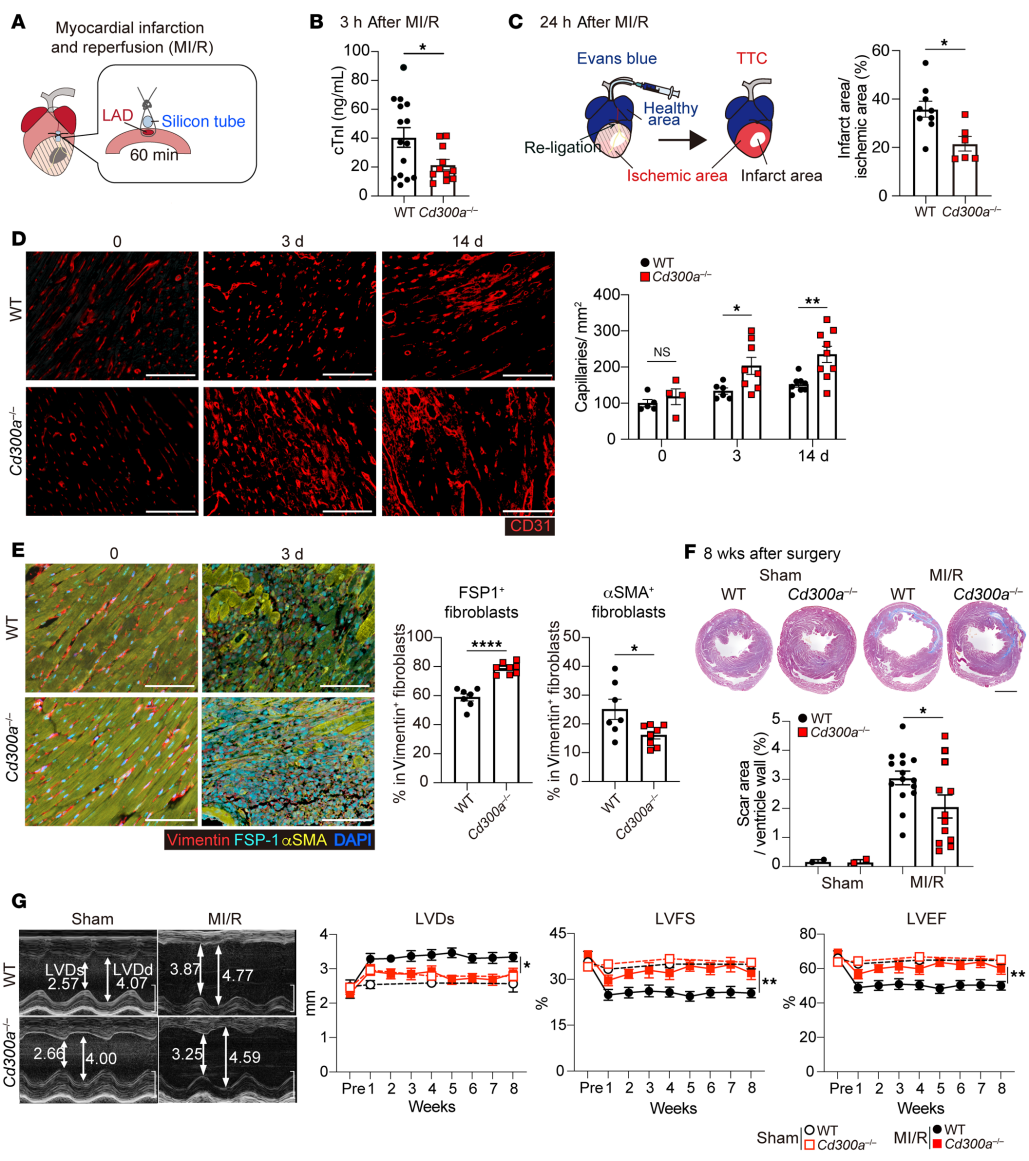
CD300a deletion ameliorates myocardial ischemia and reperfusion injury and adverse remodeling. (**A**) MI/R model. LAD, left anterior descending artery. (**B**) Plasma cTnI in *Cd300a*^–/–^ (*n* = 11) and WT mice (*n* = 15). (**C**) Evans blue and TTC staining (left) and the percentage of infarct area among ischemic area in *Cd300a*^–/–^ (*n* = 6) and WT mice (*n* = 9). (**D**) Representative immunohistochemistry (left) and quantitative data of CD31^+^ capillary density in peri-infarct area in *Cd300a*^–/–^ (*n* = 4 [day 0], 8 [day 3], and 9 [day 14]) and WT mice (*n* = 5 [day 0], 6 [day 3], and 8 [day 14]). Scale bars: 100 μm. (**E**) Representative immunohistochemistry (left) and the percentage of FSP1^+^ and αSMA^+^ fibroblasts in vimentin^+^ fibroblasts in infarct area in *Cd300a*^–/–^ (*n* = 8) and WT mice (*n* = 7). Scale bars: 100 μm. (**F**) Top: Representative Masson’s trichrome staining of the heart. Bottom: Fibrosis area in *Cd300a^–/–^* (*n* = 12 in MI/R and *n* = 2 in sham) and WT heart (*n* = 15 in MI/R and *n* = 2 in sham). Scale bar: 1 mm. (**G**) Representative echocardiography images (left) and left ventricular internal dimension in systole (LVDs), fractional shortening (LVFS), and ejection fraction (LVEF) (*n* = 2 in each group) in *Cd300a^–/–^* mice (*n* = 12) and WT mice (*n* = 15). Scale bars: 2 mm. Data are presented as mean ± SEM and pooled from more than 5 (**B**, **C**, **F**, and **G**) and 3 experiments (**D** and **E**). Dots represent independent animals. Statistical analysis was performed using unpaired Student’s *t* test (**B**, **C**, and **E**) and 2-way ANOVA (**D**, **F**, and **G**). *P < 0.05; **P < 0.01; ****P < 0.0001.

To examine whether CD300a is involved in the pathogenesis of cardiac IRI, we established a murine model of myocardial infarction and reperfusion (MI/R) by the occlusion of the left anterior descending artery for 1 hour followed by arterial reperfusion (Figure 1A). The plasma level of cardiac troponin I (cTnI), which is released by injured cardiomyocytes, was significantly lower in *Cd300a^−/−^* mice than in wild-type (WT) mice at 3 hours after MI/R (Figure 1B). Histological analysis using Evans blue and triphenyl tetrazolium chloride staining revealed that the proportion of the infarct area within the ischemic area was significantly smaller in the hearts of *Cd300a^–/–^* mice than in those of WT mice at 24 hours after MI/R (Figure 1C and Supplemental Figure 1A; supplemental material available online with this article; https://doi.org/10.1172/JCI184984DS1). Immunohistochemical analysis showed that the numbers of capillary vessels and CD31^+^ endothelial cells were significantly greater at the ischemic area in *Cd300a^−/−^* mice than in WT mice at 3 and 14 days after MI/R (Figure 1D and Supplemental Figure 1B). Moreover, the analysis demonstrated a significant increase in the proportion and the number of fibroblast-specific protein 1–expressing (FSP1-expressing) fibroblasts, which have a pro-angiogenic function in wound healing in murine and human hearts after MI (20), and a significant decrease in the proportion and the number of α-smooth muscle actin–expressing (αSMA-expressing) profibrotic fibroblasts, in the ischemic areas of cardiac tissue of *Cd300a^−/−^* mice compared with that of WT mice at 3 days after MI/R (Figure 1E and Supplemental Figure 1C), consistent with the observation of decreased fibrotic area in *Cd300a^−/−^* mice compared with WT mice at 8 weeks after MI/R (Figure 1F). Echocardiography showed that the left ventricular end-systolic dimension was significantly smaller and the left ventricular fractional shortening (LVFS) and left ventricular ejection fraction (LVEF) were significantly larger in *Cd300a^−/−^* mice than in WT mice at 4–8 weeks after MI/R (Figure 1G). To determine which cell type expressing CD300a is involved in cardiac function after MI/R, we used *Cd300a^fl/fl^Lyz2*-Cre mice and *Cd300a^fl/fl^Itgax*-Cre mice, in which CD300a expression is lacking on phagocytes, including neutrophils and both monocyte-derived and tissue-resident macrophages, and dendritic cells, respectively, in the heart. We found that LVFS and LVEF were increased in *Cd300a^fl/fl^Lyz2*-Cre mice, but not in *Cd300a^fl/fl^Itgax*-Cre mice, compared with control mice after MI/R (Supplemental Figure 1D). These results indicate that CD300a deficiency in phagocytes decreases myocardial damage and subsequent fibrosis and improves cardiac function after MI/R.

### CD300a deficiency reduces IR-induced AKI and fibrosis.

**Figure 2:**
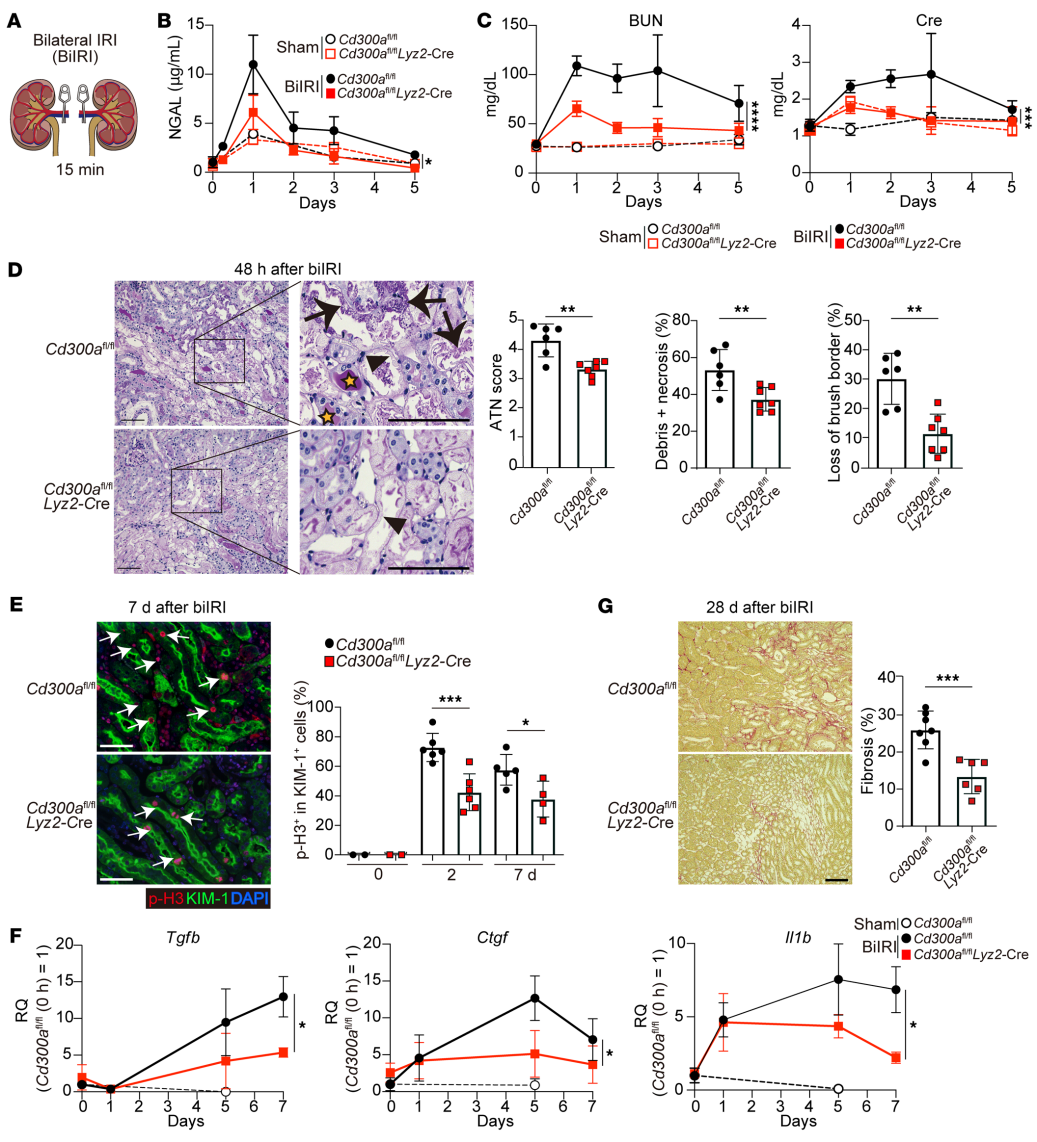
CD300a deficiency ameliorates AKI and fibrosis after biIRI. (**A**) Bilateral ischemia-reperfusion injury (biIRI) model. (**B**) Plasma NGAL in sham operation (*n* = 4 in each group) and after biIRI (*Cd300a^fl/fl^* mice, *n* = 3 [0 hours], 4 [6 hours], 13 [day 1], 7 [day 2], 6 [day 3], and 4 [day 5]; *Cd300a^fl/fl^Lyz2*-Cre mice, *n* = 3 [0 hours], 4 [6 hours], 13 [day 1], 6 [day 2], 4 [day 3], and 4 [day 5]). (**C**) Plasma BUN and Cre in sham (*n* = 4) and after biIRI (*n* = 5 [day 0], 31 [day 1], 24 [day 2], 9 [day 3], and 9 [day 5]) in each group. (**D**) Representative periodic acid–Schiff staining of kidneys after biIRI in *Cd300a^fl/fl^* and *Cd300a^fl/fl^Lyz2*-Cre mice (*n* = 6 or 7). Arrows, loss of brush border; arrowheads, necrosis; stars, debris. (**E**) Left: Representative KIM-1 and p-H3 staining of kidneys after biIRI. Right: The percentage of p-H3^+^ cells in KIM-1^+^ cells of *Cd300a^fl/fl^* (*n* = 2 [0], 6 [2 days], and 5 [7 days]) and *Cd300a^fl/fl^ Lyz2*-Cre mice (*n* = 2, 6, and 4). (**F**) *Tgfb*, *Ctgf*, and *Il1b* mRNA expression in the kidneys in sham (*n* = 2) and after biIRI (*n* = 3 [days 0, 1, and 5] and 5 [day 7] in each group). Relative quantity (RQ). (**G**) Left: Representative Sirius red staining of kidneys after biIRI. Right: Fibrosis in tubulointerstitial area in *Cd300a^fl/fl^* and *Cd300a^fl/fl^Lyz2*-Cre mice (*n* = 7 or 5). Scale bars: 100 μm (**D**, **E**, and **G**). Data are presented as mean ± SEM and pooled of 2 (**B**, **D**, **F**, and **G**), 3 (**E**), and 5 (**C**) experiments. Statistical analyses were performed using unpaired Student’s *t* test (**B**–**D**, **F**, and **G**) and 2-way ANOVA (**E**). *P < 0.05; **P < 0.01; ***P < 0.001; ****P < 0.0001.

We next analyzed AKI, in which the renal arteries were clamped bilaterally for 15 minutes followed by reperfusion (biIRI) in *Cd300a^fl/fl^* and *Cd300a^fl/fl^Lyz2-*Cre mice (Figure 2A), in which CD300a expression is lacking on the phagocytes in the kidney (Supplemental Figure 2A); this is a similar expression profile to that of *Cd300a^−/−^* mice in the heart. Levels of neutrophil gelatinase–associated lipocalin (NGAL), which is released from damaged tubular epithelial cells (21), were significantly lower in *Cd300a^fl/fl^Lyz2-*Cre mice than in *Cd300a^fl/fl^* mice 6 hours and 3 days after biIRI (Figure 2B). Blood urea nitrogen (BUN) and plasma creatinine concentrations were also significantly lower in *Cd300a^fl/fl^Lyz2-*Cre mice than in *Cd300a^fl/fl^* mice 1 and 2 days after biIRI (Figure 2C). Histological analysis of the corticomedullary junction demonstrated that acute tubular necrosis scores, as determined by the sum of scores for tubular necrosis, intratubular debris deposition, and loss of the brush border of tubular epithelial cells, were significantly lower in *Cd300a^fl/fl^Lyz2-*Cre mice than in *Cd300a^fl/fl^* mice 2 days after biIRI (Figure 2D). Moreover, although the proportion of kidney injury molecule-1–positive (KIM-1^+^) cells (i.e., damaged proximal tubular epithelial cells) was comparable between the 2 genotypes of mice 2 and 7 days after biIRI (Supplemental Figure 2B), the expression of phosphorylated histone H3 (p-H3), a marker of cell cycle arrest at G_2_/M transition, in KIM-1^+^ tubular epithelial cells was significantly lower in *Cd300a^fl/fl^Lyz2-*Cre mice than in *Cd300a^fl/fl^* mice 2 and 7 days after biIRI (Figure 2E). These p-H3–expressing tubular epithelial cells with maladaptive repair secrete profibrotic factors (22). Indeed, we found that transforming growth factor-β (*Tgfb*), *Il1b*, and connective tissue growth factor (*Ctgf*) expression was significantly lower in the kidneys of *Cd300a^fl/fl^Lyz2-*Cre mice than of *Cd300a^fl/fl^* mice 5–7 days after biIRI (Figure 2F). Furthermore, 28 days after biIRI, renal fibrosis, as analyzed by Masson’s trichrome and Sirius red staining, was significantly milder in *Cd300a^fl/fl^Lyz2-*Cre mice than in *Cd300a^fl/fl^* mice (Figure 2G and Supplemental Figure 2C).

To further analyze the CD300a involvement in the development of renal fibrosis, we used a 2-step unilateral IRI (uIRI) model (23), in which the left renal artery was clamped for 20 minutes (longer than for biIRI) and then reperfused, followed by removal of the right kidney on day 14 after uIRI (Supplemental Figure 2D), in *Cd300a^fl/fl^* and *Cd300a^fl/fl^Lyz2-*Cre mice. Plasma NGAL concentrations 24 hours after uIRI were significantly lower in *Cd300a^fl/fl^Lyz2-*Cre mice than in *Cd300a^fl/fl^* mice (Supplemental Figure 2E). Just after the removal of the right kidney on day 14 in uIRI, BUN and creatinine concentrations were significantly lower in *Cd300a^fl/fl^Lyz2-*Cre mice than in *Cd300a^fl/fl^* mice (Supplemental Figure 2F). Importantly, the degree of fibrosis on day 49 in uIRI was significantly milder in *Cd300a^fl/fl^Lyz2-*Cre mice than in *Cd300a^fl/fl^* mice (Supplemental Figure 2G). Together, these results suggest that the CD300a deficiency on phagocytes decreases the production of proinflammatory and profibrotic cytokines in the kidney, thus ameliorating AKI and subsequent fibrosis similarly to cardiac injury due to MI/R.

### CD300a deficiency enhances efferocytosis and ameliorates inflammation-associated tissue damage after cardiac and renal IRI.

**Figure 3:**
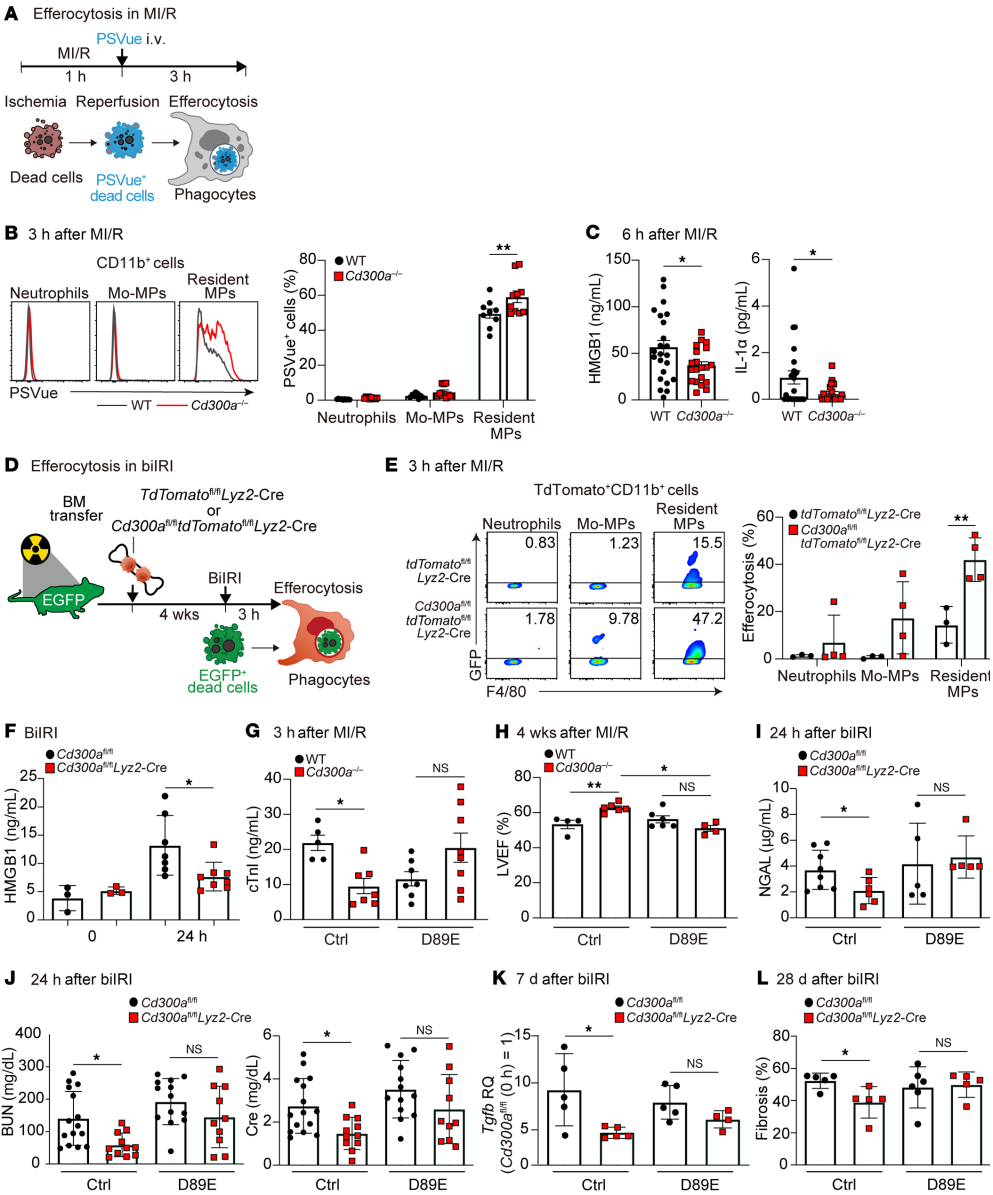
Enhanced efferocytosis reduces tissue damage and fibrosis and preserves organ function after MI/R and biIRI. (**A** and **B**) Efferocytosis of PSVue^+^ cells by CD11b^+^ myeloid cells in the heart of *Cd300a*^–/–^ (*n* = 10) and WT mice (*n* = 9). (**C**) Plasma HMGB-1 and IL-1α in *Cd300a*^–/–^ and WT mice (*n* = 23 or 24 in each group). (**D** and **E**) Efferocytosis of host-derived EGFP^+^ dead cells by donor-derived tdTomato^+^CD11b^+^ myeloid cells in the tdTomato-GFP chimeric mouse kidneys (*n* = 3 or 4 in each group). (**F**) Plasma HMGB-1 in *Cd300a^fl/fl^* and *Cd300a^fl/fl^Lyz2*-Cre mice (*n* = 3 [0 hours] and 7 or 8 [24 hours]). (**G** and **H**) Plasma cTnI and LVEF in control EPT (Ctrl)- or D89E-administered *Cd300a*^–/–^ mice (Ctrl, *n* = 7 or 6; D89E, *n* = 8 or 4) and WT mice (Ctrl, *n* = 5 or 4; D89E, *n* = 7 or 6). (**I** and **J**) Plasma NGAL and BUN and Cre in Ctrl- or D89E-administered *Cd300a^fl/fl^* (*n* = 8 or 5 for **I** and *n* = 15 or 14 for **J**) and *Cd300a^fl/fl^Lyz2*-Cre mice (*n* = 6 or 5 for **I** and *n* = 11 or 10 for **J**). (**K** and **L**) *Tgfb* expression and the fibrosis area by Masson’s trichrome staining in the kidneys of Ctrl- or D89E-administered *Cd300a^fl/fl^* (*n* = 5 in each group for **K** and *n* = 5 or 6 for **L**) and *Cd300a^fl/fl^Lyz2*-Cre mice (*n* = 5 or 4 for **K** and *n* = 5 in each group for **L**). Data are presented as mean ± SEM and pooled of 2 (**E**, **I**, **K**, and **L**), 3 (**B**, **C**, and **F**–**H**), and 5 (**J**) experiments. Two-way ANOVA (**B** and **E**–**L**) and unpaired Student’s *t* test (**C**). *P < 0.05; **P < 0.01.

To examine how CD300a regulates myocardial damage and subsequent fibrosis after MI/R, we determined the expression of CD300a on myeloid cells in cardiac tissue after MI/R. CD300a expression was confirmed on neutrophils (CD11b^+^Ly6G^+^), monocyte-derived macrophages (CD11b^+^Ly6G^−^Ly6C^+^), and tissue-resident macrophages (CD11b^+^Ly6G^−^Ly6C^−^CD64^+^) in the cardiac tissue of naive WT mice, and this expression — particularly on the tissue-resident macrophages — was upregulated at 3 days after MI/R (Supplemental Figure 3A). To examine whether CD300a was involved in the efferocytosis after MI/R, we intravenously administered PSVue-643, a near-infrared fluorescent probe for the detection of PS-expressing dead cells, to WT and *Cd300a^−/−^* mice immediately after MI/R and used flow cytometry to evaluate efferocytosis by myeloid cells (Figure 3A). *Cd300a^−/−^* mice showed a significantly higher proportion of tissue-resident macrophages expressing PSVue-643 fluorescence than did WT mice at 3 hours after MI/R; in both types of mice, neither neutrophils nor inflammatory monocytes showed PSVue-643 fluorescence (Figure 3B), indicating that *Cd300a^−/−^* tissue-resident macrophages showed enhanced efferocytosis after MI/R. In line with these results, *Cd300a^−/−^* mice showed lower plasma levels of DAMPs such as HMGB-1 and IL-1α in the coronal sinus and had significantly fewer tissue-infiltrating inflammatory cells, including neutrophils and monocyte-derived macrophages, than WT mice at 6 hours and 3 days, respectively, after MI/R (Figure 3C and Supplemental Figure 3B).

To investigate the role of CD300a in efferocytosis after IRI in the kidney, we transferred bone marrow (BM) cells from td*Tomato^fl/fl^Lyz2-*Cre or *Cd300a^fl/fl^*td*Tomato^fl/fl^Lyz2-*Cre mice into irradiated enhanced green fluorescent protein–expressing (EGFP-expressing) R26GRR mice. Four weeks later, these mice, in which the transferred phagocytes expressed tdTomato fluorescence, were subjected to biIRI (Figure 3D). Flow cytometric analyses demonstrated that although each myeloid cell population was comparable between the 2 genotypes of mice 3 hours after biIRI (Supplemental Figure 3C), the population of EGFP-expressing resident macrophages derived from *Cd300a^fl/fl^*td*Tomato^fl/fl^Lyz2-*Cre mice was significantly larger than that derived from td*Tomato^fl/fl^Lyz2-*Cre mice 3 hours after biIRI (Figure 3E). Furthermore, laser scanning confocal microscopy analysis revealed that the EGFP signal was detected in the cytoplasm of the tdTomato^+^ myeloid cells, indicating that these myeloid cells engulfed host-derived dead tissues, and the phagocytic index, as determined by the numbers of EGFP-expressing macrophages and the magnitude of EGFP signals detected in each macrophage, was larger in *Cd300a^fl/fl^*td*Tomato^fl/fl^Lyz2-*Cre macrophages than in td*Tomato^fl/fl^Lyz2-*Cre macrophages (Supplemental Figure 3D). These results indicate that mice deficient in CD300a on tissue-resident macrophages showed enhanced efferocytosis in the kidney after biIRI, as was also observed after MI/R. In line with these results, *Cd300a^fl/fl^Lyz2-*Cre mice showed lower levels of HMGB-1 in the plasma and expression of the proinflammatory cytokines *Il6*, *Tnfa*, and *Il1b*, and a smaller number of tissue-infiltrating inflammatory cells, including monocyte-derived macrophages and neutrophils, than *Cd300a^fl/fl^* mice after biIRI (Figure 3F and Supplemental Figure 3, E and F).

Milk-fat globulin protein E8 (MFG-E8) mediates efferocytosis via cross-linking PS on dead cells to α_v_β_3_ integrin on macrophages. However, MFG-E8 mutated at residue 89 (D89E-MFG-E8), which binds to PS, but not α_v_β_3_, inhibits PS receptor–mediated efferocytosis (24). D89E-MFG-E8 or a control protein (EPT-MFG-E8; mutant MFG-E8 with altered binding regions for PS) was injected i.v. into WT or *Cd300a^−/−^* mice immediately after MI/R. Whereas EPT-MFG-E8 administration into mice resulted in significantly lower plasma levels of cTnI and a significantly higher LVEF in *Cd300a^−/−^* mice than in WT mice, D89E-MFG-E8 administration resulted in comparable plasma levels of cTnI and a comparable LVEF in the 2 genotypes of mice (Figure 3, G and H). D89E-MFG-E8 administration also increased plasma NGAL, BUN, and creatinine concentrations in *Cd300a^fl/fl^Lyz2-*Cre mice to levels comparable to those seen in *Cd300a^fl/fl^* mice 24 hours and 48 hours after biIRI (Figure 3, I and J, and Supplemental Figure 3, G and H). Moreover, the *Tgfb* expression in the kidney and the degree of renal fibrosis, as analyzed by histological analysis using Masson’s trichrome staining, in *Cd300a^fl/fl^Lyz2-*Cre mice were increased to a level seen in *Cd300a^fl/fl^* mice after D89E-MFG-E8 treatment 28 days after biIRI (Figure 3, K and L). Together, these results suggest that increased efferocytosis due to CD300a deficiency on tissue-resident macrophages reduced tissue damage accompanying the reduction of release of DAMPs from dead cells and tissue-infiltrating cells and ameliorated cardiac and renal injuries and the following aberrant remodeling after IR through a similar mechanism.

### CD300a deficiency upregulates pro-angiogenic and antifibrotic gene expression in SiglecF^lo^ neutrophils.

**Figure 4:**
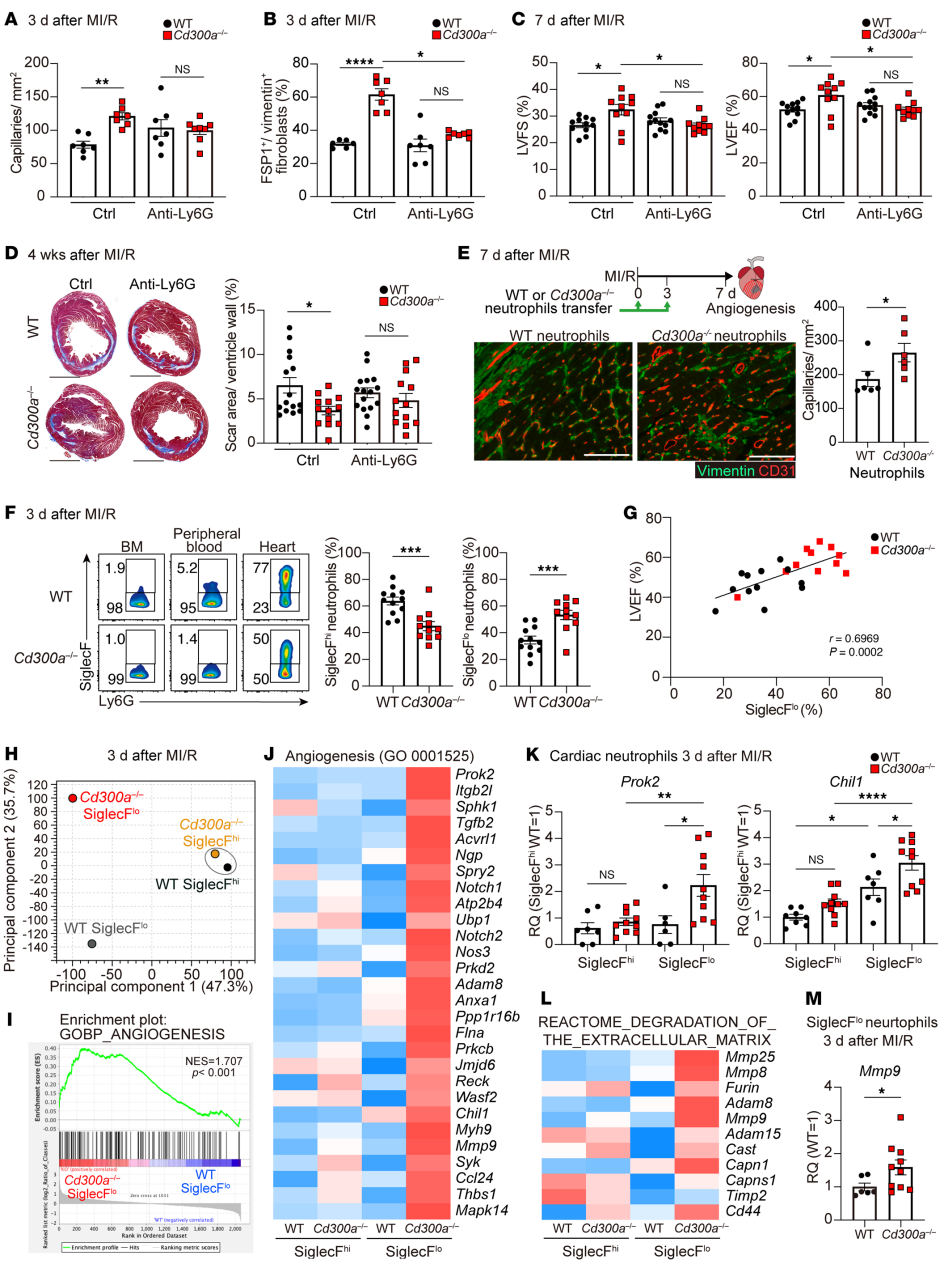
CD300a deficiency upregulates pro-angiogenic and antifibrotic genes in SiglecF^lo^ neutrophils. (**A**–**D**) CD31^+^ capillary density in peri-infarct area (**A**), the percentage of FSP1^+^ cells (**B**), LVFS and LVEF (**C**), and the percentage of scar areas (**D**) in isotype control (Ctrl)– or anti-Ly6G mAb–administered *Cd300a*^–/–^ and WT mice (Ctrl, *n* = 6–8 [**A** and **B**], 10–12 [**C**], and 13–16 [**D**]). Scale bars: 1 mm. (**E**) CD31^+^ capillary density in the peri-infarct area in the heart of mice into which WT or *Cd300a*^–/–^ neutrophils were transferred. Scale bars: 100 μm. (**F**) Flow cytometric analysis of SiglecF^hi^ and SiglecF^lo^ neutrophils in the cardiac tissue in WT (*n* = 12) and *Cd300a*^–/–^ (*n* = 11) mice. (**G**) The correlation between LVEF and the percentages of SiglecF^lo^ neutrophils. (**H**) Principal component analysis plot of RNA expression of SiglecF^hi^ and SiglecF^lo^ neutrophils in the cardiac tissue from WT and *Cd300a*^–/–^ mice (*n* = 8 in each group). (**I**) GSEA profiles of enrichment gene sets associated with angiogenesis in *Cd300a*^–/–^ and WT SiglecF^lo^ neutrophils. (**J**) Heatmap of enriched genes associated with angiogenesis. (**K**) *Prok2* and *Chil1* mRNA expression in SiglecF^hi^ and SiglecF^lo^ neutrophils in cardiac tissue in *Cd300a*^–/–^ and WT mice (*n* = 10 or 7). (**L**) Heatmap of enriched genes associated with degradation of the extracellular matrix. (**M**) *Mmp9* mRNA expression in SiglecF^lo^ neutrophils in the cardiac tissue in *Cd300a*^–/–^ and WT mice (*n* = 7 or 10). Data are presented as mean ± SEM and pooled from 2 (**E**–**G**) and more than 5 (**A**–**D**, **K**, and **M**) experiments. Two-way ANOVA (**A**–**D** and **K**) and unpaired Student’s *t* test (**E**, **F**, and **M**). *P < 0.05; **P < 0.01; ***P < 0.001; ****P < 0.0001.

Since CD300a is expressed on neutrophils as well as macrophages, we analyzed the role of CD300a on neutrophils in the pathogenesis of cardiac function after MI/R by depleting neutrophils. WT and *Cd300a^−/−^* mice were injected i.v. with a depleting anti-Ly6G mAb on days –1, 0, and 1 after MI/R, and immune cells in the heart were analyzed for cell surface expression of Gr-1 by an anti–Gr-1 mAb and intracellular expression of Ly6G by the anti-Ly6G mAb by flow cytometry. On day 3 after MI/R, both cell surface Gr-1 and intracellular Ly6G were not detected in the neutrophil population in the heart (Supplemental Figure 4A). In contrast, the monocyte-derived macrophage population (CD11b^+^Ly6C^+^Gr-1^–^Ly6G^–^) was not altered by injection of the anti-Ly6G mAb (Supplemental Figure 4A), indicating that neutrophils but not macrophages were depleted in the heart on day 3 after MI/R. We found that the anti-Ly6G mAb treatment decreased capillary density and FSP1-expressing fibroblasts at 3 days after MI/R in *Cd300a^−/−^* mice to a level comparable to that in WT mice (Figure 4, A and B, and Supplemental Figure 4B). Furthermore, compared with WT mice, control mAb injection increased LVFS and LVEF at day 7 and decreased scar area at 4 weeks after MI/R in *Cd300a^−/−^* mice, but administration of anti-Ly6G mAb abolished these differences between *Cd300a^−/−^* and WT mice (Figure 4, C and D), suggesting that CD300a on neutrophils is involved in angiogenesis, the development of fibrosis, and cardiac function after MI/R. To confirm this idea, we transferred *Cd300a^−/−^* or WT neutrophils into WT mice immediately and 3 days after MI/R. We found that mice that received *Cd300a^−/−^* neutrophils showed higher capillary density in the cardiac tissue than those that received WT neutrophils (Figure 4E). Together, these results suggest that *Cd300a^−/−^* neutrophils promote angiogenesis and cardiac function after MI/R.

Recent studies revealed that SiglecF^hi^ neutrophils are increased in the cardiac tissue after myocardial infarction in mice (9, 10). Whereas most neutrophils in the BM and peripheral blood of WT and *Cd300a^−/−^* mice were found to express no, or a low level of, SiglecF (SiglecF^lo^), those in the post-MI/R cardiac tissue of both genotypes of mice were found to highly express SiglecF (SiglecF^hi^) (Figure 4F). However, *Cd300a^−/−^* mice exhibited a significantly lower proportion of SiglecF^hi^ neutrophils and a significantly higher proportion of SiglecF^lo^ neutrophils in injured cardiac tissue than in WT mice (Figure 4F). Furthermore, when LVEF at day 3 after MI/R was used as a measure, a larger proportion of SiglecF^lo^ neutrophils was positively correlated with better cardiac function (Figure 4G), suggesting that SiglecF^lo^ neutrophils play an important role in angiogenesis and cardiac function after MI/R.

To further characterize *Cd300a^−/−^* neutrophils involved in the pathology of MI/R injury, we used RNA sequence analysis to compare the gene expression profiles of the 2 neutrophil subsets purified from cardiac tissue from the 2 genotypes of mice at day 3 after MI/R. Whereas the SiglecF^hi^ neutrophils from both genotypes of mice showed a similar gene expression profile, the gene expression profiles of the SiglecF^lo^ neutrophils differed (Figure 4H and Supplemental Figure 4, C and D). The most prominent genes upregulated in *Cd300a^−/−^* SiglecF^lo^ neutrophils were associated with angiogenesis (Figure 4, I and J, and Supplemental Figure 4E). For example, the mRNA expression of *Prok2* and *Chil1*, which both encode pro-angiogenic factors, was upregulated more in *Cd300a^−/−^* SiglecF^lo^ neutrophils than in WT SiglecF^lo^ neutrophils (Figure 4K). However, WT and *Cd300a^−/−^* SiglecF^lo^ neutrophils from the peripheral blood showed comparable expression levels of these mRNAs (Supplemental Figure 4F). In addition, genes associated with collagen degradation in the extracellular matrix, including genes encoding members of the matrix metalloproteinase (MMP) family and the ADAM metallopeptidase family, were upregulated in *Cd300a^−/−^* SiglecF^lo^ neutrophils (Figure 4, L and M). These results suggest that CD300a regulates the expression of genes encoding pro-angiogenic and antifibrotic factors in SiglecF^lo^ neutrophils.

In contrast, SiglecF^hi^ neutrophils showed upregulation of genes associated with inflammatory responses and fibrosis (Supplemental Figure 4, G–J). For example, the mRNA expression levels of genes encoding proinflammatory cytokines, such as tumor necrosis factor-α (*Tnf*), interleukin-1β (*Il1b*), *Il6*, and the profibrotic factor *Tgfb1*, were significantly higher in SiglecF^hi^ neutrophils than in SiglecF^lo^ neutrophils, although the expression levels of those genes were comparable between WT and *Cd300a^−/−^* neutrophils (Supplemental Figure 4, K and L). These results indicate that SiglecF^hi^ neutrophils play pathogenic roles by exacerbating inflammation and fibrosis, regardless of CD300a expression, after MI/R.

Notably, we also found that the SiglecF^hi^ neutrophil population was smaller, and the SiglecF^lo^ neutrophil population was larger, in *Cd300a^fl/fl^Lyz2-*Cre mice than in *Cd300a^fl/fl^* mice in the kidney 14 days after biIRI (Supplemental Figure 4M), suggesting a functional role of CD300a on neutrophils in the kidney after biIRI that is similar to that observed in the heart after MI/R.

### CD300a inhibits STAT3 phosphorylation in SiglecF^lo^ neutrophils.

**Figure 5:**
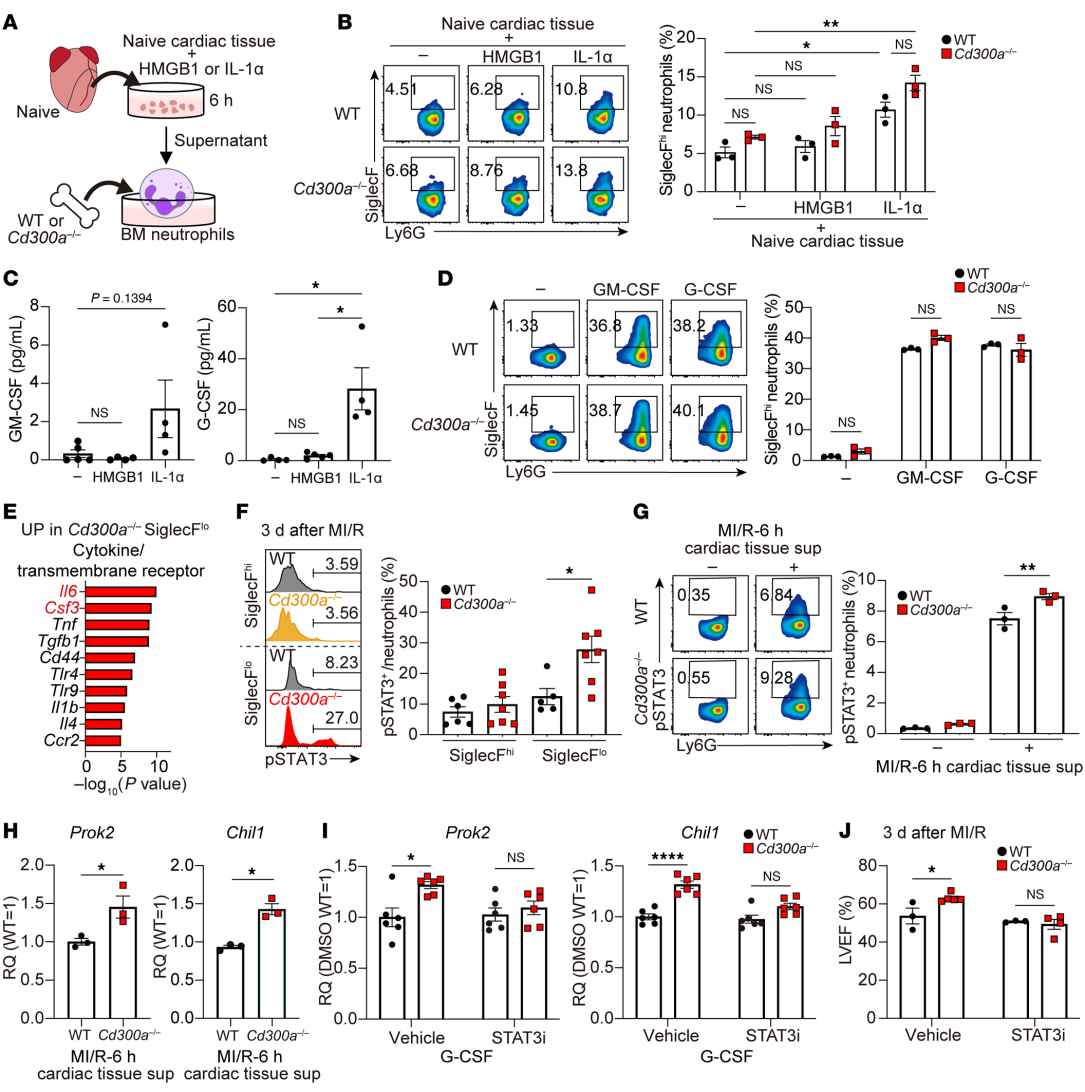
CD300a suppresses STAT3 phosphorylation in SiglecF^lo^ neutrophils. (**A**–**C**) WT or *Cd300a^−/−^* BM neutrophils were cultured for 2 days in the presence of the culture supernatant of the naive cardiac tissue, which had been stimulated or not with HMGB-1 or IL-1α (**A**) and analyzed for SiglecF expression by flow cytometry (**B**). (**C**) The GM-CSF and G-CSF levels in the culture supernatant of the cardiac tissue after stimulation with HMGB-1 or IL-1α. (**D**) Flow cytometric analysis of SiglecF expression on WT or *Cd300a^−/−^* BM neutrophils after stimulation with GM-CSF or G-CSF. (**E**) Ingenuity Pathway Analysis based on differentially expressed genes from *Cd300a*^–/–^ and WT SiglecF^lo^ neutrophils. (**F** and **G**) Flow cytometric analysis of phosphorylated STAT3 (p-STAT3) in neutrophils of the cardiac tissue of *Cd300a*^–/–^ (*n* = 7) and WT (*n* = 6) mice (**F**) and WT or *Cd300a*^–/–^ BM neutrophils stimulated with or without the cardiac tissue supernatant (**G**). (**H** and **I**) *Prok2* and *Chil1* mRNA expression in BM neutrophils of WT or *Cd300a*^–/–^ mice stimulated with the cardiac tissue supernatant after MI/R (**H**) or with G-CSF together with STAT3 inhibitor (STAT3i) or control vehicle (Vehicle) (**I**). (**J**) LVEF after MI/R in *Cd300a*^–/–^ (vehicle, *n* = 5; STAT3i, *n* = 4) and WT mice (*n* = 3 in each group) treated with STAT3i or vehicle. Data are presented as mean ± SEM, representative of 2 experiments (**B**, **D**, **G**, and **H**) and pooled from 2 (**C**, **F**, and **I**) and 4 (**J**) experiments. One-way (**B**–**D**) and 2-way ANOVA (**F**, **G**, **I**, and **J**) and unpaired Student’s *t* test (**H**). *P < 0.05; **P < 0.01; ****P < 0.0001.

To examine how *Cd300a*^–/–^ mice had a lower proportion of SiglecF^hi^ neutrophils than did WT mice, we stimulated WT or *Cd300a^−/−^* BM neutrophils with the supernatant from a suspension of WT mouse cardiac tissue collected before, or 6 hours after, MI/R (Supplemental Figure 5A). The supernatant of cardiac tissue after MI/R, but not that from naive heart, induced the generation of SiglecF^hi^ neutrophils, even though the WT and *Cd300a^−/−^* BM neutrophil populations contained comparable proportions of SiglecF^hi^ neutrophils (Supplemental Figure 5B). This suggests that the transition of SiglecF^lo^ to SiglecF^hi^ neutrophils was induced by extrinsic factors in the heart after MI/R. We hypothesized that DAMPs, such as HMGB-1 or IL-1α, after MI/R (Figure 3C) may induce the generation of SiglecF^hi^ neutrophils in the injured heart. However, neither HMGB-1 nor IL-1α had any effect on the generation of SiglecF^hi^ neutrophils when neutrophils purified from the BM were stimulated with DAMPs alone (Supplemental Figure 5C). By contrast, SiglecF^hi^ neutrophils were generated when BM neutrophils were stimulated with the culture supernatant of the naive cardiac tissues after stimulation with IL-1α, but not HMGB-1 (Figure 5, A and B), suggesting that IL-1α indirectly induces the generation of SiglecF^hi^ neutrophils by stimulating cardiac cells rather than neutrophils.

IL-1α induces the production of granulocyte-macrophage colony-stimulating factor (GM-CSF) and granulocyte colony-stimulating factor (G-CSF) by fibroblasts in arthritis, arthrofibrosis, and skin fibrosis (25, 26). Similarly, we found here that stimulation of WT cardiac cells with IL-1α induced the production of GM-CSF and G-CSF (Figure 5C). Stimulation of purified BM neutrophils with GM-CSF or G-CSF induced the generation of SiglecF^hi^ neutrophils, with the WT and *Cd300a^−/−^* BM neutrophil populations containing comparable proportions of SiglecF^hi^ neutrophils (Figure 5D). Since IL-1α expression was lower in *Cd300a^−/−^* mice than in WT mice (Figure 3C) because of enhanced efferocytosis in the former after MI/R (Figure 3B), these results suggest that the lower release of IL-1α in *Cd300a^−/−^* mice resulted in reduced production of G-CSF and GM-CSF, which in turn resulted in the generation of fewer SiglecF^hi^ neutrophils.

To elucidate how CD300a regulates gene expression in SiglecF^lo^ neutrophils, the signals regulated by CD300a in SiglecF^lo^ neutrophils were predicted by Ingenuity Pathway Analysis using gene sets enriched in *Cd300a^−/−^* SiglecF^lo^ neutrophils compared with those in WT SiglecF^lo^ neutrophils, as determined using RNA-Seq data. Because CD300a is localized on the cell surface and suppresses proximal activating signals derived from nearby localized receptors, we focused on signals from cell surface receptors. The top 2 upstream candidates were the genes encoding IL-6 and G-CSF receptor–mediating signaling pathway (Figure 5E), respectively, which activate STAT3 in neutrophils and promote angiogenesis (27, 28). Indeed, the genes upregulated in *Cd300a^−/−^* SiglecF^lo^ neutrophils were enriched in the STAT3 signaling pathway (Supplemental Figure 5D). *Cd300a^−/−^* SiglecF^lo^ neutrophils exhibited a significantly higher proportion of phosphorylated STAT3 than did WT SiglecF^lo^ neutrophils and WT SiglecF^hi^ neutrophils in the cardiac tissue on day 3 after MI/R (Figure 5F). Stimulation of BM neutrophils with a supernatant from cardiac tissue after MI/R resulted in significantly greater STAT3 phosphorylation (Figure 5G) and upregulation of the pro-angiogenic genes *Prok2* and *Chil1* in *Cd300a^−/−^* neutrophils compared with WT neutrophils (Figure 5H), suggesting that CD300a suppresses the expression of pro-angiogenic and antifibrotic genes by inhibiting STAT3 activation in SiglecF^lo^ neutrophils in cardiac tissue after MI/R. Among the candidate CD300a-targeting signals predicted by the ingenuity pathway analysis, G-CSF stimulation, but not IL-6, increased the phosphorylation of STAT3 and the expression of *Prok2* and *Chil1* in *Cd300a^−/−^* BM neutrophils, whereas a STAT3 inhibitor (STAT3i) reduced their expression to levels comparable to those in WT neutrophils (Figure 5I and Supplemental Figure 5, E and F), indicating that CD300a inhibits the G-CSF/STAT3 axis for the expression of *Prok2* and *Chil1* in *Cd300a^−/−^* BM neutrophils in vitro. Moreover, STAT3i abolished the improved LVEF in *Cd300a^−/−^* mice at day 3 after MI/R (Figure 5J). Taken together, although other cell types in addition to *Cd300a^−/−^* BM neutrophils were also involved in the effect of STAT3i in vivo, these results suggest that pro-angiogenic genes are induced via the G-CSF/STAT3 axis, which is inhibited by CD300a in SiglecF^lo^ neutrophils and attenuates cardiac dysfunction after MI/R.

### CD300a blockade with a neutralizing antibody ameliorates cardiac and renal IRI and adverse remodeling.

**Figure 6:**
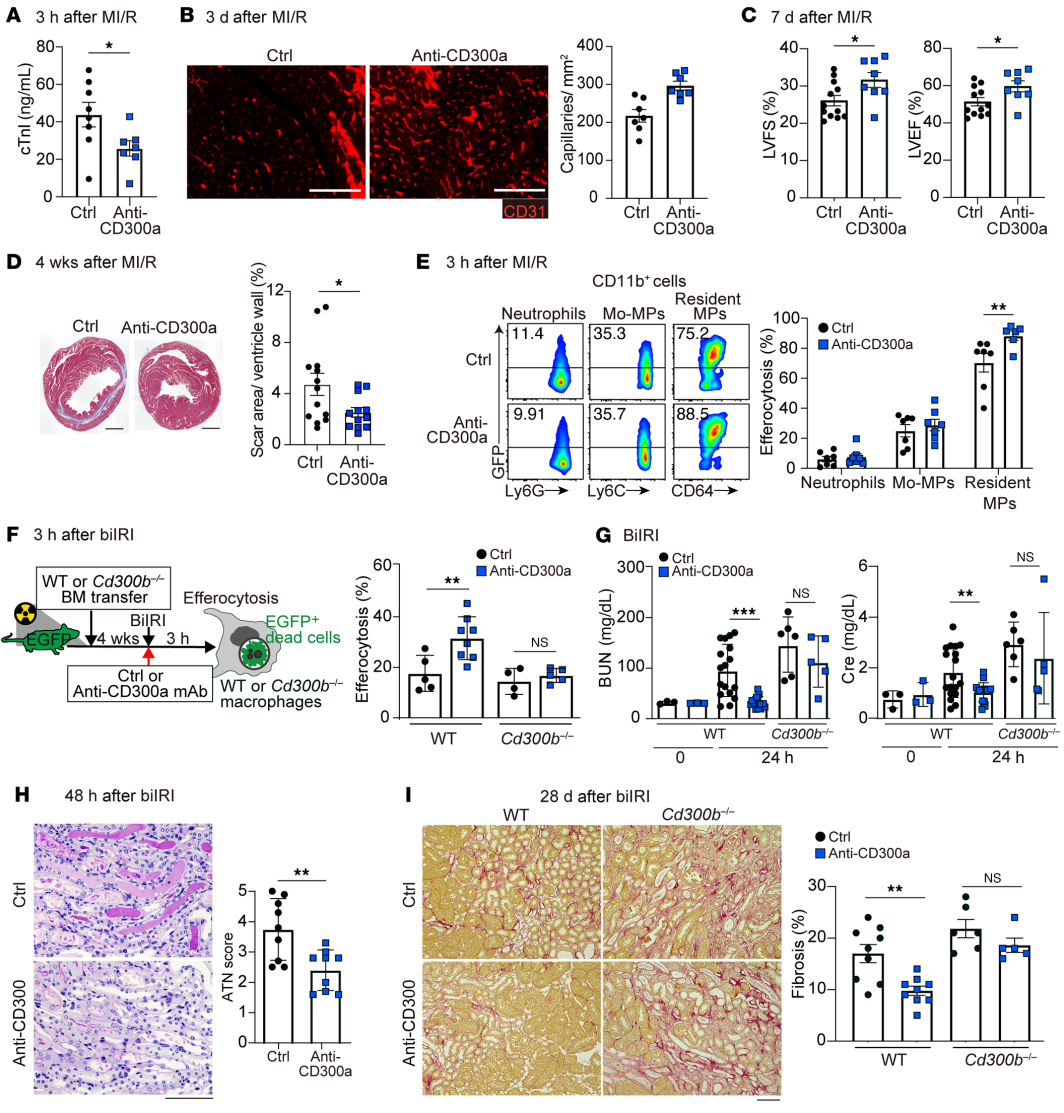
Anti-CD300a mAb ameliorates tissue injury and adverse remodeling. (**A**) Plasma cTnI in anti-CD300a mAb– or Ctrl-administered mice (*n* = 7 or 8). (**B**) CD31^+^ capillary density in peri-infarct area in anti-CD300a mAb– or Ctrl-administered mice (*n* = 7). (**C**) LVFS and LVEF in anti-CD300a mAb– or Ctrl-administered mice (*n* = 7 or 5). (**D**) Masson’s trichrome staining and the fibrosis area in the ventricle in anti-CD300a mAb– or Ctrl-administered mice (*n* = 10 or 11). (**E**) Efferocytosis of GFP^+^ dead cells by myeloid cell subpopulations in the cardiac tissue of αMHC-GFP mice given anti-CD300a mAb or Ctrl (*n* = 6 or 7). (**F**) Generation of BM chimeric R26GRR mice (GFP^+^ mice) and efferocytosis of host-derived GFP^+^ dead cells by resident macrophages of the chimeric mice given an anti-CD300a mAb (*n* = 8 or 5) or Ctrl (*n* = 5 or 4). (**G**) Plasma BUN and Cre in WT and *Cd300b^–/–^* mice given anti-CD300a mAb or Ctrl (WT, *n* = 3 or 17; *Cd300b^–/–^*, *n* = 5 or 6). (**H**) Periodic acid–Schiff staining of the kidney and acute tubular necrosis (ATN) score in mice given anti-CD300a mAb or Ctrl (*n* = 9 in each group). (**I**) Sirius red staining and the fibrosis area in WT and *Cd300b^–/–^* mice given anti-CD300a mAb or Ctrl (WT, *n* = 8; *Cd300b^–/–^*, *n* = 5 or 6). Scale bars: 100 μm (**B**, **H**, and **I**) and 1 mm (**D**). Data are presented as mean ± SEM and pooled from 2 (**A**–**D**, **F**, **H**, and **I**) and 5 (**E** and **G**) experiments. Student’s *t* test (**A**–**D** and **H**) and 2-way ANOVA (**E**–**G** and **I**). *P < 0.05; **P < 0.01; ***P < 0.001.

To determine whether CD300a blockade ameliorates cardiac and renal IRI, we injected an anti-CD300a neutralizing mAb or control mAb i.v. into WT mice immediately after MI/R and 2 hours before biIRI and 2-step uIRI. Administration of this antibody did not affect the percentage of neutrophil and macrophage populations in the kidney 1 and 7 days after biIRI (Supplemental Figure 6A) (29). Consistent with our earlier observations in *Cd300a^−/−^* mice (Figure 1), treatment with the anti-CD300a mAb reduced the plasma cTnI level, increased angiogenesis, improved LVFS and LVEF, and reduced fibrosis compared with control antibody injection after MI/R (Figure 6, A–D). To determine whether these effects of anti-CD300a mAb were caused by enhanced efferocytosis, we used αMHC-GFP mice, in which only mature cardiomyocytes express GFP (30). Treatment with the anti-CD300a mAb enhanced efferocytosis by resident macrophages, but not neutrophils and monocyte-derived macrophages, as determined by GFP expression by flow cytometry, compared with that of the control antibody (Figure 6E).

To analyze the effect of the anti-CD300a mAb on IRI in the kidney, we used the BM chimeric mice reconstituted with WT or *Cd300b^−/−^* BM cells (Figure 3D). These chimeric mice received i.v. the anti-CD300a mAb or control mAb at 2 hours before biIRI. Similarly to efferocytosis by the resident macrophages from *Cd300a^fl/fl^*td*Tomato^fl/fl^Lyz2-*Cre mice (Figure 3E), efferocytosis by resident macrophages was enhanced (Figure 6F), and plasma BUN and creatinine concentrations and acute tubular necrosis scores on histology at 24 hours and 48 hours, respectively, after biIRI were significantly lower, in mice treated with the anti-CD300a mAb than in those treated with the control mAb (Figure 6, G and H). Moreover, renal fibrosis at 28 days after biIRI was significantly milder in mice treated with the anti-CD300a mAb than in those treated with the control antibody (Figure 6I). Using a 2-step uIRI, during the 49-day observation period, plasma BUN and creatinine concentrations were significantly lower, and the degree of renal fibrosis was significantly milder, in mice treated with the anti-CD300a mAb than in mice treated with the control antibody (Supplemental Figure 6, B and C). Moreover, treatment with the anti-CD300a mAb, even 3 hours after uIRI, significantly improved renal function (Supplemental Figure 6D).

CD300a on macrophages suppresses CD300b-mediated efferocytosis by inhibiting the CD300b-associated DAP12 activation in vitro and neurological damage after middle cerebral artery occlusion in mice (19). We examined whether this is also the case in IRI in the kidney. As expected, the anti-CD300a mAb had no effect on efferocytosis, plasma BUN and creatinine concentration levels, and renal fibrosis in CD300b-deficient mice (Figure 6, F, G, and I). These results suggest that CD300a blockade by the neutralizing anti-CD300a mAb enhanced CD300b-mediated efferocytosis and ameliorated both tissue injury and fibrosis after cardiac and renal IR.

### A humanized anti-CD300A mAb ameliorates renal IRI in humanized mice.

**Figure 7:**
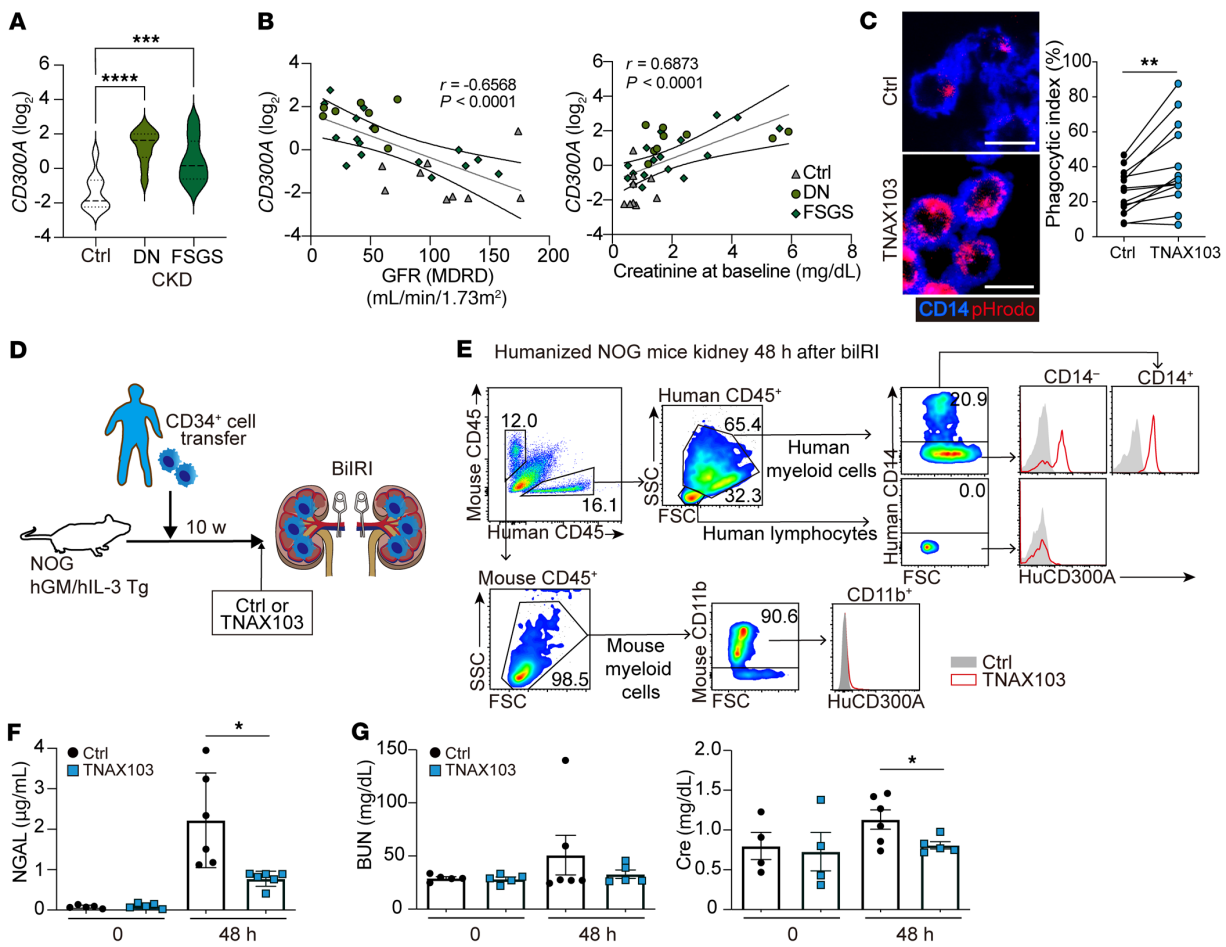
Blockade of human CD300A enhances efferocytosis and ameliorates acute renal injury after biIRI. (**A** and **B**) A public gene expression dataset of patients with CKD of diabetic nephropathy (DN) (*n* = 9) and focal segmental glomerulosclerosis (FSGS) (*n* = 19). *CD300A* expression in comparison with the healthy control group (*n* = 10) (**A**) and Spearman’s correlation analysis between the expression of *CD300A* and renal function of glomerular filtration rate (GFR) and creatinine (**B**). MDRD, Modification of Diet in Renal Disease formula. (**C**) Efferocytosis of pHrodo-labeled dead Jurkat cells by human monocytes from 13 donors in the presence of control mAb (Ctrl) and a humanized anti-CD300A mAb (TNAX103). Left: Representative laser scanning confocal microscopy image. Right: Phagocytic index. Scale bars: 100 µm. (**D**) Schematic diagram of the generation of the humanized mice by transferring of human CD34^+^ cells to NOG mice with transgenic expression of human GM-CSF and IL-3. These mice were given Ctrl or a humanized anti-CD300A mAb (TNAX103) 2 hours before biIRI. (**E**) Flow cytometric analysis of CD300A expression on human and mouse CD45^+^ cells in the kidney in the humanized mice after biIRI. (**F** and **G**) Plasma NGAL (**F**) and BUN and Cre (**G**) before (0) and 48 hours after biIRI in the humanized mice that had been injected i.v. with TNAX103 mAb or Ctrl (*n* = 5 or 6, respectively). Data are presented as mean ± SEM and pooled from 2 (**F** and **G**) and 3 (**C**) experiments. Student’s *t* test (**C**) and 2-way ANOVA (**F** and **G**). *P < 0.05; **P < 0.01; ***P < 0.001; ****P < 0.0001.

Using a public dataset (Nephroseq v5, http://v5.nephroseq.org) of gene expression profiles in CKD patients, *CD300A* expression was significantly higher in patients with CKD due to diabetic nephropathy and focal segmental glomerulosclerosis than in control patients (Figure 7A). In addition, Spearman’s correlation analysis demonstrated that the expression level of *CD300A* was negatively correlated with glomerular filtration rate but positively correlated with the plasma concentration of creatinine (Figure 7B). These results suggest the possibility that CD300A is involved in the transition to CKD after AKI in humans.

We generated a neutralizing mouse anti–human CD300A mAb, named TX113, that binds to transfectants stably expressing CD300A (31). We further generated a humanized neutralizing mAb based on the complementarity-determining regions of TX113 with the human IgG1-Fc portion with a mutated amino acid at residues 234 and 235 from leucine to alanine (named TNAX103) (32). We cocultured peripheral blood monocytes with dead Jurkat cells, which had been labeled with a pH-sensitive fluorescent dye (pHrodo), in the presence of TNAX103 or control mAb, and we analyzed efferocytosis by confocal laser scanning microscopy. In these experiments, the phagocytic index of monocytes was significantly higher in the presence of TNAX103 than in the presence of the control mAb (Figure 7C). To clarify the role of TNAX103 in the development of AKI after IRI, human CD34^+^ stem cells were transplanted into NOD/SCID mice deficient in the common γ chain of the IL-2 receptor (NOG mice) with transgenic expression of human GM-CSF and IL-3 (named NOG-EXL mice) (Figure 7D). Ten weeks later, we confirmed the engraftment of human CD45^+^ hematopoietic cells expressing CD300A in the peripheral blood of naive mice (Supplemental Figure 7) and CD14^+^ myeloid cells expressing CD300A in the kidney 48 hours after biIRI (Figure 7E). These mice were given TNAX103 or a control mAb 2 hours before biIRI. Forty-eight hours after biIRI, plasma NGAL and creatinine concentrations were significantly lower in TNAX103-treated than in control mAb–treated mice (Figure 7, F and G). These results suggest that TNAX103 is potentially useful for the treatment of ischemic organ diseases such as AMI and AKI and the prevention of CHF and CKD.

## Discussion

Here, we showed that CD300a deficiency ameliorates tissue injury, dysfunction, and fibrosis in the heart and kidney after IR by enhancing efferocytosis by tissue-resident macrophages and decreasing the release of dead-cell-derived DAMPs such as IL-1α and/or HMGB-1. We previously reported that CD300a blockade ameliorated acute ischemic stroke by IR in mice (19). IRI in the heart and kidney differs from that in the brain in that CD300a blockade enhanced efferocytosis by tissue-resident macrophages in the heart and kidney, but not by inflammatory monocyte-derived macrophages in the brain. The most important finding in the heart and kidney, which was not observed in the brain, is that CD300a blockade also suppressed fibrosis after IR. Recent studies have reported that SiglecF^hi^ neutrophils are elicited at the injured tissue by DAMPs and have a pathogenic function to induce fibrosis by producing profibrotic or proinflammatory cytokines such as TGF-β, TNF-α, and IL-1β, and also by directly producing collagen I in a mouse model of CKD (12). SiglecF^hi^ neutrophils originate from the BM, and after migration from the peripheral blood to injured tissues, they acquire their characteristic gene expression profile associated with pathogenesis of inflammation (10, 33). Recent studies have demonstrated that DAMPs or inflammation-associated cytokines such as GM-CSF and TGF-β induce the generation of pathogenic SiglecF^hi^ neutrophils in asthma and CKD (11, 12). Here, we found that one of the DAMPs, IL-1α, stimulated G-CSF and GM-CSF production in the cardiac tissue, likely by fibroblasts (25, 26), and that these cytokines induced the generation of SiglecF^hi^ neutrophils after MI/R. Since we showed here that IL-1α induced the generation of SiglecF^hi^ neutrophils at comparable levels in WT and *Cd300a^−/−^* mice, the decrease of SiglecF^hi^ neutrophils in *Cd300a^−/−^* mice after MI/R may have been caused by a decreased amount of IL-1α in the infarct lesion in the heart. We also showed that the SiglecF^hi^ neutrophil population was lower in *Cd300a^fl/fl^Lyz2-*Cre mice than in *Cd300a^fl/fl^* mice in the kidney after biIRI, suggesting a similar mechanism of tissue injury and fibrosis in the kidney as compared with the heart. Regardless, CD300a deficiency decreased the pathogenic SiglecF^hi^ neutrophil population, contributing in part to the reduction of tissue injury, fibrosis, and organ dysfunction after MI/R and likely after renal IR.

DAMPs such as HMGB-1 are also known to induce endoplasmic reticulum stress and G_2_/M arrest of tubular epithelial cells (6, 22). These cells with G_2_/M arrest express *Tgfb* and *Ctgf* through an interaction between cyclin G1 and cyclin-dependent kinase 5, as well as via the histone deacetylase 9 (HDAC9)/STAT1 signaling pathway, causing CKD with fibrosis (34, 35). Here, we showed that HMGB-1 was decreased at 24 hours after IR, and that this was followed by a decrease in the number of p-H3^+^ tubular cells with G_2_/M arrest 2 days after IR and a decrease in *Tgfb*, *Ctgf*, and *Il1b* expression in the kidney 5–7 days after IR. These results suggest that the decrease in DAMPs due to enhanced efferocytosis may have resulted in the reduction of maladaptive tubular repair in addition to the decrease in SiglecF^hi^ neutrophils, thereby suppressing the production of fibrosis-inducing cytokines and preventing fibrosis.

In contrast to the functional characteristics of SiglecF^hi^ neutrophils, those of SiglecF^lo^ neutrophils remain to be fully determined. Here, we uncovered that SiglecF^lo^ neutrophils express genes encoding angiogenic and antifibrotic cytokines such as Prok2, Chil1, and MMP9. Notably, we showed that CD300a regulates the production of these cytokines by SiglecF^lo^ neutrophils by inhibiting STAT3 phosphorylation. Thus, CD300a deficiency promotes the production of these cytokines, thereby contributing in part to the reduction of adverse remodeling with fibrosis and cardiac dysfunction after MI/R. Together, these results suggest that CD300a deficiency prevents the development of CHF after MI/R via both SiglecF^hi^ and SiglecF^lo^ neutrophils: CD300a deficiency in tissue-resident macrophages increased efferocytosis and thereby decreased DAMPs, which directly reduced inflammation-associated tissue injury and indirectly reduced it via decreasing the generation of pathogenic SiglecF^hi^ neutrophils. In addition, decreasing DAMPs via enhanced efferocytosis increased the proportion of SiglecF^lo^ neutrophils with proangiogenic and antifibrotic characteristics, resulting in prevention of fibrosis and subsequent CHF. Since the SiglecF^hi^ and SiglecF^lo^ neutrophils were lower and higher, respectively, in *Cd300a^fl/fl^Lyz2-*Cre mice than in *Cd300a^fl/fl^* mice in the kidney after biIR, CD300a on neutrophils, as well as tissue-resident macrophages, likely plays an important role in the development of AKI and subsequent renal fibrosis.

We showed that a humanized anti-CD300A mAb enhanced efferocytosis by human monocytes in vitro. To study the role of efferocytosis in the development of IRI in humans, we used humanized mice. Treatment of the humanized mice with the humanized anti-CD300A mAb improved AKI after IR. Because the humanized anti-CD300A mAb specifically binds to human CD300A but not mouse CD300a, these results suggest that the mAb enhanced efferocytosis of mouse dead cells by tissue-resident human macrophages and suppressed the inflammation induced by DAMPs, preventing tissue damage such as that to tubular epithelial cells and glomeruli in the mouse kidney. This AKI model using humanized mice is useful for studying the role of humanized CD300A mAb in efferocytosis via human macrophages in vivo. Our results suggest that the anti-CD300A mAb can be used to treat ischemic organ diseases, including AMI, AKI, and others, and for the prevention of the transition to CHF and CKD.

## Methods

### Sex as a biological variable.

Our study examined only male mice to avoid hormonal cycle–related variability. It is unknown whether the findings are relevant to female mice.

### Mice.

C57BL/6J mice were purchased from Clea Japan. *Cd300a^−/−^* (17), *Cd300a^fl/fl^*, *Lyz2*-Cre, *Cd300a^fl/fl^Lyz2*-Cre (19), *Cd300b^−/−^* (19), R26GRR (*Rosa26^CAG-EGFP/tdsRed^*) (36), td*Tomato^fl/fl^* [*Gt*(*ROSA*)*26Sor^tm14(CAG-tdTomato)Hze^*] (37), and αMHC-GFP mice (30) on the C57BL/6 background were described previously. NOG mice expressing transgenic human GM-CSF and IL-3 (NOG-EXL), into which human cord blood CD34^+^ cells had been transferred, were purchased from In-Vivo Science. The chimerism rate of human cells in peripheral blood was greater than 26%.

### Ischemia and reperfusion models in the heart and kidney.

Male mice (8–12 weeks old, 23–29 g) were used to induce MI/R (38), biIRI, and 2-stage uIRI. Mice were anesthetized by intraperitoneal administration of 0.2 mg/kg of medetomidine, 0.8 mg/kg of midazolam, and 0.5 mg/kg of butorphanol. For the induction of MI/R, mice were intubated and ventilated at 140 respirations/min and 18 cm H_2_O of maximal peak pressure by ventilator (Mouse Ventilator Minivent Type 845, Harvard Apparatus). Anesthesia was maintained with 2.0%–2.5% isoflurane with a heating pad during the surgery. A fourth intercostal thoracotomy was performed to expose the heart. A silicon tube (PE-10 tube) and the left anterior descending artery (LAD) were ligated with a 7-0 silk suture, and after 60 minutes, the silicon tube and the suture were removed to allow reperfusion. For neutrophil depletion, anti-Ly6G mAb or isotype control mAb was administered intraperitoneally on days –1, 0, 1, 3, and 5 after MI/R. For inhibition of STAT3, 5 mg/kg STAT3 inhibitor (C188-9; S8605, Selleckchem) or vehicle (DMSO) was administered intraperitoneally daily from 0 to 3 days after MI/R. For induction of the biIRI, both renal pedicles were clamped for 15 minutes. For 2-stage uIRI, the left renal pedicle was clamped for 20 minutes on day 0, and the right kidney was removed on day14 (39). During the kidney procedures, mice were placed on a 43°C heat pad.

### ELISA.

cTnI, NGAL, and HMGB-1 in the plasma were measured using a cardiac Troponin-I ELISA kit (CTNI-1-HSP, Life Diagnostics), mouse NGAL ELISA kit (KE10045, Proteintech), and HMGB-1 ELISA kit II (326078738, Shino-Test Corp.), respectively, in accordance with the manufacturers’ instructions.

### Infarct and ischemia area measurements.

Twenty-four hours after MI/R, the heart was removed, and aortic cannulation was performed to inject 1 mL of saline to remove the remaining intracavitary blood. The LAD was re-ligated at the same location as in the ischemia induction, and then 0.5 mL of 1% Evans blue dye was injected into the aorta through the indwelling cannula to stain the non-ischemic area. Then, the heart was placed in an Eppendorf tube, frozen on dry ice, and sectioned into 1 mm short-axis slices. The slices were incubated with 1% 2,3,5-triphenyl tetrazolium chloride (TTC) solution at 37°C for 15 minutes to stain viable myocardium as red and visualize the infarct area as white. The slices were fixed in 4% paraformaldehyde at room temperature. The non-ischemic area stained with Evans blue, the ischemic area stained with TTC (red area), and the infarct area (white area) were measured digitally using ImageJ software (NIH).

### Tissue preparation for histology.

Hearts and kidneys were fixed with 10% formalin-buffered solution and embedded in paraffin. The heart and kidney slices, 3 μm thick, were stained by Masson’s trichrome, periodic acid–Schiff, or Sirius red, and analyzed using a digital BZ-X700 light and fluorescent microscope and BZ-X analysis software (Keyence). The percentage of fibrosis area was calculated as the ratio of fibrosis area to the whole ventricle wall or kidney in each section. To analyze acute tubular necrosis score in the kidney, the percentage of tubules that displayed cell necrosis, loss of brush border, cast formation, and tubule dilatation was counted as follows: 0, none; 1, <10%; 2, 11% to 25%; 3, 26% to 45%; 4, 46% to 75%; and 5, >76%. For the fibrosis score, 10 fields in ×200 magnification were reviewed in each slide.

### Immunohistochemistry.

For multiplex immunohistochemistry (IHC), antibody staining and signal amplification were performed in accordance with the manufacturer’s protocol (Opal 7-Color Manual IHC Kit, PerkinElmer) (40). Slide sections were incubated at room temperature for 1 hour or at 4°C overnight with primary antibodies in antibody diluent (Dako): CD31 (PECAM-1) (D8V9E) XP at ×200 dilution (Cell Signaling Technology, 77699); anti-vimentin antibody (Abcam, EPR3776) at ×500 dilution (Abcam, ab92547); anti–α-smooth muscle actin (anti-αSMA) antibody at ×100 dilution (Abcam, ab5694); anti-FSP1/S100A4 antibody at ×200 dilution (Sigma-Aldrich); anti–mouse Ly6G mAb (1A8) at ×10,000 dilution (Bio X Cell, BE0075-1); anti–KIM-1 antibody at ×200 dilution (R&D, AF1817); anti–p-H3 antibody at ×200 dilution (Abcam, ab5176). Slides were washed twice with Tris-buffered saline containing 0.05% Tween 20 (TBST) for 3 minutes and incubated with 2–3 drops of anti-mouse/rabbit Opal Polymer horseradish peroxidase (HRP) (ARH1001EA, PerkinElmer) or anti-rat IgG HRP-linked species-specific whole antibody (Cytiva, NA935) for 30 minutes at room temperature. Tyramide signal amplification (TSA; PerkinElmer) was performed with the Opal 570 (vimentin, CD31, and KIM-1), Opal 480 (FSP1, Ly6G, and p-H3), or Opal 690 (αSMA) for 10 minutes at room temperature. Multiplex TSA experiments were performed by repeating of staining cycles in series, with an antigen retrieval step each time, and then counterstained with the nuclear counterstain DAPI (VectaShield, Vector Laboratories). A Mantra Snap 1.0.3 (PerkinElmer) fluorescence microscope was used with epifluorescence filters of FITC, TRITC, Cy3, Texas red, Cy5, and DAPI. Each Opal fluorochrome and DAPI were unmixed using mono fluorescence-stained slides, and multi-stained images were analyzed with tissue segmentation, cell segmentation, and positive score by inForm Advanced Image Analysis software (inForm 2.6, PerkinElmer). At least 3 fields at ×20 magnification containing ischemic area were analyzed for each sample. For the analysis of CD31^+^ capillary density, blinded quantification of CD31^+^ capillaries was performed using the taken IHC images with ×20 magnification.

### Echocardiography.

Transthoracic echocardiography was performed using the Vevo 2100 imaging system (Fujifilm Visual Sonics) under anesthesia with 1.5% isoflurane. Long-axis B-mode and M-mode images were used for measurement of the echocardiography parameters, including left ventricular (LV) end-diastolic dimension (LVDd) and end-systolic dimension (LVDs). The Teichholz formula (volume = 7LVD3/[2.4 + LVD]) was used to calculate the LV end-diastolic volume (LVEDV) and LV end-systolic volume (LVESV). LV ejection fraction (LVEF) and fractional shortening (LVFS), measures of LV systolic function, were calculated using the following formula: LVEF (%) = (LVEDV – LVESV)/LVEDV × 100; LVFS (%) = ([LVDd – LVDs]/LVDd) × 100.

### Cell preparation.

After mice were perfused with 20 mL PBS under deep anesthesia, their hearts and kidneys were collected and minced with scissors into about 0.5 mm pieces in 5 mL of RPMI 1640 with 10% fetal bovine serum (FBS). The hearts were digested with collagenase II (200 U/mL; Worthington Biochemical) and DNase I (60 U/mL; Worthington Biochemical) at 37°C for 30 minutes with agitation on a rotator. Cells were separated using the gentleMACS Dissociator (Miltenyi Biotec) according to the manufacturer’s instructions and washed with PBS supplemented with 1% FBS. The kidneys were digested using a tumor dissociation kit (Miltenyi Biotec) according to the manufacturer’s instructions, followed by lysing of erythrocytes using the ammonium chloride–potassium (ACK) lysis buffer (Thermo Fisher Scientific). For the isolation of renal macrophages, renal cells were isolated with CD11b^+^ microbeads (Miltenyi Biotec) using LS columns (Miltenyi Biotec). BM neutrophils were isolated by negative selection using the Neutrophil Isolation Kit (Miltenyi Biotec), as previously described (41). Human monocytes were purified from the peripheral blood with the EasySep Human Monocyte Isolation Kit (STEMCELL Technologies).

### Antibodies and flow cytometry.

Cells were incubated with Zombie NIR (BioLegend), Zombie Violet (BioLegend), or propidium iodide to exclude dead cells and anti–mouse CD16/32 (clone 2.4G2, BD Biosciences) or a human FcR blocking reagent (Miltenyi Biotec) in a staining medium (PBS containing 2% FBS and 0.01% sodium azide) for 15 minutes to block the Fcγ receptors. Cells were then stained with the following antibodies for 30 minutes on ice. The fluorochrome-conjugated anti-mouse antibodies specific to CD45.2 (phycoerythrin [PE]-Cy7, clone 104), CD45.2 (BV421, clone 104), CD11b (BV711, clone M1/70), Ly6G (allophycocyanin [APC], clone 1A8), Ly6G (FITC, clone 1A8), Ly6C (PE, clone HK1.4), Ly6C (APC and Alexa Fluor 700, clone HK1.4), CD64 (BV605, clone X54-5/7.1), CD64 (PE, clone X54-5/7.1), CD11C (PE-Cy7, clone N418), I-A/I-E (BV510, clone M5/114.15.2), CD3e (FITC, clone 145-2C11), CD19 (FITC, clone 1D3/CD19), NK1.1 (FITC, clone PK136), BV421-streptavidin, and PE-streptavidin were purchased from BioLegend. Fluorochrome-conjugated anti-mouse antibodies specific to SiglecF (BV421 and PE, clone E50-2440), Gr-1 (PE, clone RB6-8C5), CD31 (Alexa Fluor 647, clone 390), and p-STAT3 (pY705, Alexa Fluor 488, clone 4/p-STAT3) were purchased from BD Biosciences, and CD11b (APC-Cy7, clone M1/70) was from TOMBO. Fluorochrome-conjugated anti-human antibodies specific to CD16 (PE, clone 3G8), CD56 (BV421, clone B159), CD19 (FITC, clone HIB19), CD4 (FITC, clone L200), and CD3 (PE, clone HIT3a) were purchased from BD Pharmingen, CD4 (V500, clone RPA-T4) was from BD Biosciences, CD14 (FITC and APC, clone Tuk4) was from Invitrogen, and CD45 (BV421, clone 2D1) and CD8 (FITC, clone RPA-T8) were from BioLegend. Anti–mouse CD300a mAb (EX42) (29) and humanized anti–human CD300A mAb (TNAX103) (31, 32) were generated in our laboratory, as described previously (42). Recombinant human IgG1 (LALA-PG, N/A-CP149) was purchased from Bio X Cell.

All samples were analyzed using the LSR Fortessa flow cytometer (BD Biosciences) and FlowJo software (Tree Star). Doublets were excluded by gating out the population defined by forward scatter and side scatter in the flow cytometry in all the experiments. For p-STAT3 expression analysis, cells were fixed in Phosflow Lyse/Fix buffer (BD Biosciences) for 10 minutes at 37°C and permeabilized with BD Phosflow Perm Buffer III for 30 minutes on ice before staining with anti–p-STAT3 (pY705) antibody.

### Biochemical analysis.

Plasma BUN and creatinine were measured using FUJI DRI-CHEM SLIDE BUN-PIII and CRE-PIII, respectively, according to the manufacturer’s instructions.

### Quantitative reverse transcriptase PCR.

Total RNA was extracted from renal cells 1, 5, and 7 days after biIRI or from neutrophils isolated from the heart 3 days after MI/R using FACSAria II (BD Biosciences). RNA was extracted using ISOGEN (Nippon Gene). cDNA was synthesized using a High-Capacity cDNA Reverse Transcription Kit (Applied Biosystems). Quantitative reverse transcriptase PCR was performed using Power SYBR Green PCR Master Mix (Applied Biosystems) and an ABI 7500 sequence detector (Applied Biosystems). Normalization of quantitative real-time PCR was performed based on the gene encoding β-actin. The sequences of primers were as follows: β-actin forward, 5′-ACTGTCGAGTCGCGTCCA-3′; β-actin reverse, 5′-GCAGCGATATCGTCATCCAT-3′; *Tgfb1* forward, 5′-TGACGTCACTGGAGTTGTACGG-3′; *Tgfb1* reverse, 5′-GGTTCATGTCATGGATGGTGC-3′; *Prok2* forward, 5′-GCCCCGCTACTGCTACTTC-3′; *Prok2* reverse, 5′-CCGCACTGAGAGTCCTTGTC-3′; *Chil1* forward, 5′-GTACAAGCTGGTCTGCTACTTC-3′; *Chil1* reverse, 5′-ATGTGCTAAGCATGTTGTCGC-3′; *Il1b* forward, 5′-ACTCAACTGTGAAATGCCACC-3′; *Il1b* reverse, 5′-TGATACTGCCTGCCTGAAGC-3′; *Mmp9* forward, 5′-ACGACATAGACGGCATCCAGTATC-3′; *Mmp9* reverse, 5′-AGGTATAGTGGGACACATAGTGGG-3′; *Il6* forward, 5′-AGTTGCCTTCTTGGGACTGA-3′; *Il6* reverse, 5′-TCCACGATTTCCCAGAGAAC-3′; *Tnf* forward, 5′-AGTTGCCTTCTTGGGACTGA-3′; *Tnf* reverse, 5′-TCCACGATTTCCCAGAGAAC-3′; *Ctgf* forward, 5′-GTGGAATATTGCCGGTGCA-3′; *Ctgf* reverse, 5′-CCATTGAAGCATCTTGGTTCG-3′.

### Cytometric bead array analysis.

To measure the amount of IL-1α, the plasma was collected from the coronary sinus at 6 hours after MI/R and analyzed by cytometric bead array (CBA) according to the manufacturer’s instructions (BD Biosciences) followed by CBA analysis FCAP software (BD Biosciences).

### In vivo blocking of PS interaction with CD300a.

D89E-MFG-E8 and the control protein (EPT-MFG-E8) (100 mg of each protein), which were generated as previously described (19), were administered i.v. 10 minutes before MI/R or biIRI.

### RNA sequence.

Single-cell suspension was prepared from the heart, and SiglecF^hi^ and SiglecF^lo^ neutrophils (CD45.2^+^CD11b^+^Ly6G^+^) were isolated using FACSAria II (BD Biosciences). In each experiment, more than 2 × 10^4^ neutrophils from 8 mice were pooled. RNA was extracted and purified using ISOGEN (Nippon Gene) and NEBNext Poly(A) mRNA Magnetic Isolation Module (New England Biolabs), respectively. Sequencing libraries of purified mRNA were prepared using the NEBNext Ultra II RNA Library Prep Kit (New England Biolabs). The synthesized libraries were checked for yield using an Agilent High Sensitivity DNA Kit (Agilent Technologies). The libraries were sequenced with a NextSeq500/550 v2.5 Kit (20024906, Illumina) for 75 cycles to obtain more than 20 million reads as paired-end reads in each sample. CLC Genomics Workbench 20.0 software (QIAGEN) was used to calculate transcripts per million (TPM) and fold change and to generate principal component analysis. The genes with TPM of more than 10 were used in the analysis. Heatmapper (http://www.heatmapper.ca/) was used to make the heatmap; R was used to make the volcano plot. Gene Ontology analysis (biological processes) of differentially expressed genes was performed using the Database for Annotation, Visualization and Integrated Discovery (DAVID; v2023q2, https://davidbioinformatics.nih.gov). Gene set enrichment analysis (GSEA) was performed using GSEA version 4.3.2 (https://www.gsea-msigdb.org/gsea/index.jsp). RNA sequence analysis of healthy and CKD kidneys was performed using the Nephroseq database (www.nephroseq.org; University of Michigan, Ann Arbor, MI, USA).

### Preparation of the supernatant from cardiac tissue.

The hearts were collected, and the luminal blood was washed out with RPMI 1640 with 10% FBS. The hearts were then crushed with frosted-edge glass slides and stimulated or not with HMGB-1 (1 μg/mL) or IL-1α (10 ng/mL) for 6 hours, and the supernatant was collected.

### Adoptive transfer of neutrophils.

BM-derived neutrophils (1 × 10^6^ cells) from WT or *Cd300a^−/−^* mice were injected i.v. into WT mice on days 0 and 3 after MI/R as previously described (12). Cardiac tissue was collected on day 7 after MI/R to perform IHC analysis.

### BM chimeric mice.

BM chimeric mice were generated as described previously (19). In brief, R26GRR mice at 4–5 weeks old were irradiated with 5 Gy twice. BM cells (1 × 10^7^) from td*Tomato^fl/fl^Lyz2*-Cre, *Cd300a^fl/fl^*td*Tomato^fl/fl^Lyz2*-Cre, wild-type, or *Cd300b^−/−^* mice were injected into the tail veins of irradiated recipients immediately after the final irradiation. Four weeks after BM cell transfer, these mice were subjected to renal IR.

### Efferocytosis assay.

Dead Jurkat cells were generated using 1 μM staurosporine (Wako Fujifilm) and stained with 100 ng/mL pHrodo-SE (Invitrogen) for 30 minutes at room temperature. Human monocytes (2 × 10^5^) were incubated with anti–human CD300A mAbs (10 mg/mL) for 120 minutes at 37°C and cocultured with dead Jurkat cells (4 × 10^5^) for 120 minutes at 37°C. Efferocytosis was analyzed by confocal laser scanning microscopy (Fluoview FV10i, Olympus). Digital images of randomly selected fields under confocal microscopy were captured, and more than 100 cells that engulfed dead cells were analyzed. The phagocytic index was calculated according to the following formula (43): phagocytic index = (total number of engulfed cells/total number of counted macrophages) × (number of macrophages containing engulfed cells/total number of counted macrophages) × 100.

### Statistics.

Statistical analyses were performed using Prism 9 or 10 (GraphPad Software). Results are presented as mean ± SEM. *P* values less than 0.05 were considered statistically significant. The unpaired or paired Student’s *t* test (2-tailed) or 1- or 2-way ANOVA tests were performed to compare more than 2 groups along with Bonferroni’s multiple comparisons test.

### Study approval.

All animal experiments were approved by the University of Tsukuba Animal Resource Center (approved number: 23-332) and performed in accordance with the guidelines of the animal ethics committee of the University of Tsukuba Animal Resource Center (Tsukuba, Japan).

### Data availability.

The RNA-Seq data were deposited in the NCBI’s Gene Expression Omnibus database (GEO GSE263749) and are publicly available as of the date of publication. All data generated in this study are available in the Supporting Data Values file.

## Author contributions

CNO and AS conceptualized the study. NN, HK, CNO, SF, XN, HL, FA, JL, and YX conducted the investigation. NN, HK, SF, FA, JL, and K Tajiri performed animal studies. NN, HK, CNO, XN, HL, and TS performed visualization. TS and MI contributed to methodology. NN, HK, and CNO wrote the manuscript. CNO, K Tabuchi, KS, and AS reviewed and edited the manuscript. CNO and AS supervised and acquired funding for the study.

## Supplementary Material

Supplemental data

Supporting data values

## Version 1 07/24/2025

In-Press Preview

## Version 2 10/01/2025

Electronic publication
