# Supplementary material for: Immunoreceptor CD300a regulates ischemic tissue damage and adverse remodeling in the mouse heart and kidney
Source: J Clin Invest. 2025 Jul 24;135(19):e184984. doi: 10.1172/JCI184984 (PMC12483560; doi:10.1172/JCI184984)
Supplement: Supplemental data [file jci-135-184984-s136.pdf]

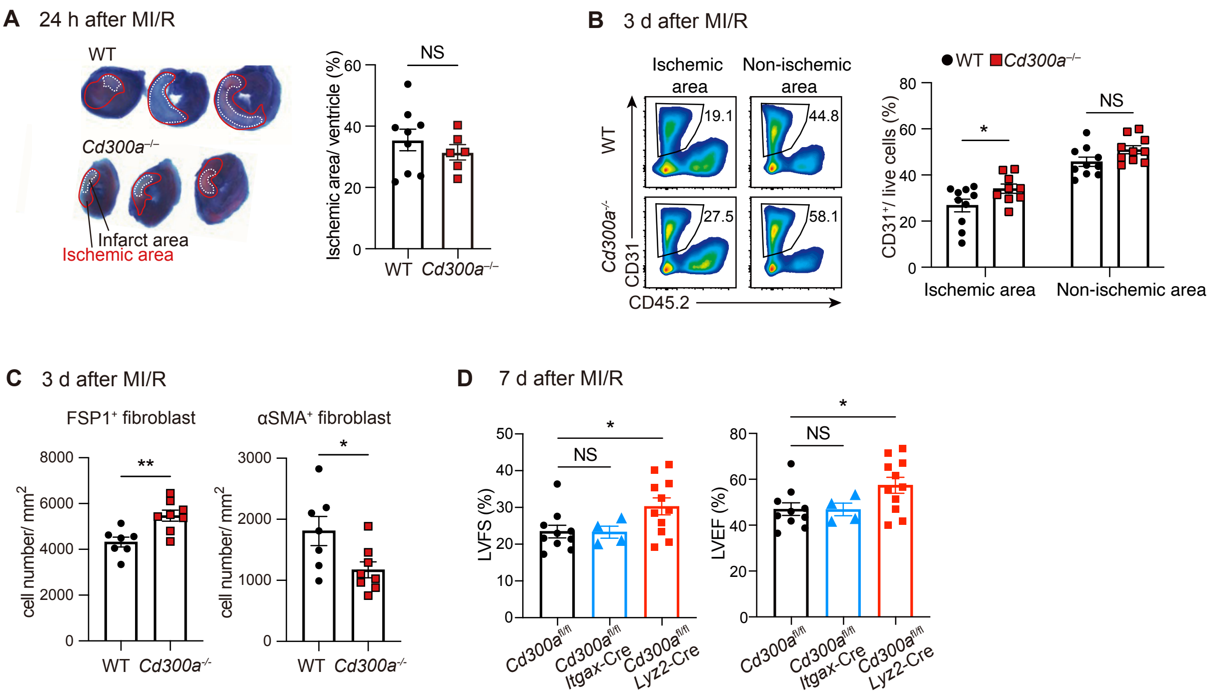

Fig. S1. CD300a deletion ameliorates myocardial ischemia and reperfusion injury and adverse remodeling.

(**A**) Representative images of Evans blue and TTC staining (left) and the percentage of ischemic area in *Cd300a*$^{-/-}$ and WT mice 24 h after MI/R (right). The blue, red, and white areas indicate non-ischemic (healthy), ischemic, and infarct areas, respectively. (**B**) Flow cytometric analysis of CD31^{+} vascular endothelial cell in the ischemic area and non-ischemic area in *Cd300a*$^{-/-}$and WT mice (n = 10 in each group) 3 days after MI/R. (**C**) Cell numbers of vimentin^{+}FSP1^{+} (left) and vimentin^{+}αSMA^{+} (right) fibroblast in the infarct area 3 days after MI/R in *Cd300a*$^{-/-}$ mice (n = 8) and WT mice (n = 7). (**D**) Left ventricular fractional shortening (LVFS) and ejection fraction (LVEF) analyzed by echocardiography in *Cd300a*$^{fl/fl}$ (n = 10), *Cd300a*$^{fl/fl}$*Itgax*-Cre (n = 4) and *Cd300a*$^{fl/fl}$*Lyz2*-Cre (n = 11) mice 8 weeks after MI/R. Data are presented as means ± SEM and pooled of more than 5 (A and D) and 3 (B and C) experiments. Statistical analysis was performed using unpaired Student's *t*-test (A and C) and two-way ANOVA (B and D). *, $P < 0.05$; **, $P < 0.01$; NS, not significant.

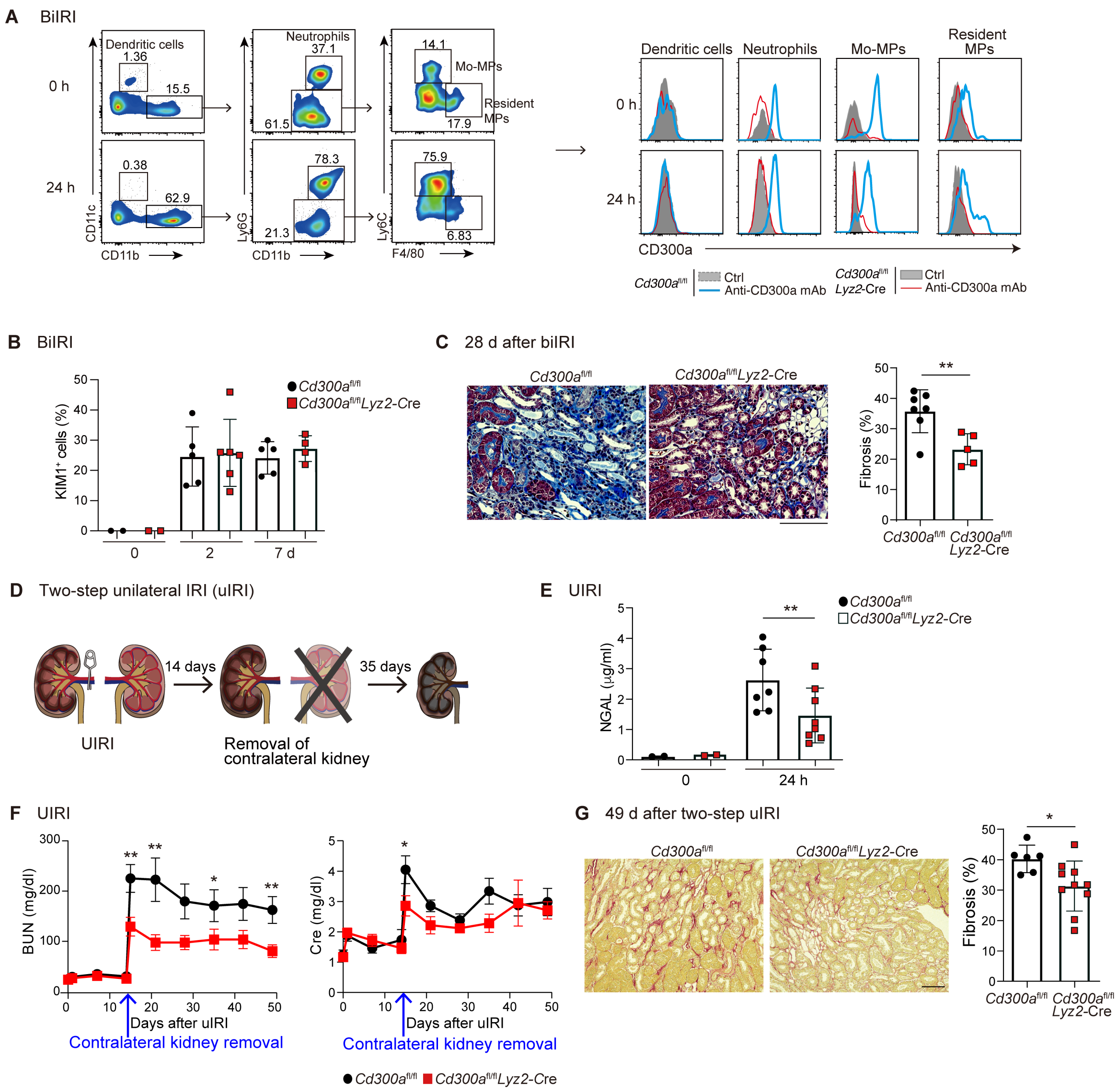

Fig. S2. **CD300a deficiency ameliorates acute kidney injury and fibrosis after uIRI.** (**A**) Gating strategy and representative flow cytometry analysis of the expression of CD300a on dendritic cells, neutrophils, monocyte-derived macrophages (Mo-MPs) and resident macrophages (resident MPs) in the kidney of *Cd300a*$^{\text{fl/fl}}$ and *Cd300a*$^{\text{fl/fl}}$*Lyz2*-Cre mice before (0 h) and 24 h after biIRI. (**B**) The percentage of KIM-1^{+} cells in the kidney before (0) and 2 and 7 d after biIRI in *Cd300a*$^{\text{fl/fl}}$ (n = 2, 5 and 5, respectively) and *Cd300a*$^{\text{fl/fl}}$ *Lyz2*-Cre mice (n=2, 6 and 4, respectively). (**C**) Representative Masson-trichrome staining of the kidney 28 d after biIRI (left). Fibrosis in tubulointerstitial area in *Cd300a*$^{\text{fl/fl}}$ (n = 7) and *Cd300a*$^{\text{fl/fl}}$ *Lyz2*-Cre (n = 6) mice (right). (**D**) Schematic diagram of two-step unilateral ischemia-reperfusion injury model (uIRI). (**E**) Plasma NGAL before (0) and 24 h after uIRI in *Cd300a*$^{\text{fl/fl}}$ (n = 2 and 7, respectively) and *Cd300a*$^{\text{fl/fl}}$*Lyz2*-Cre (n = 2 and 8, respectively) mice. (**F**) Plasma BUN and Cre before (0) and 1, 7, 14, 21, 28, 35, 42, and 49 d after uIRI in *Cd300a*$^{\text{fl/fl}}$ and *Cd300a*$^{\text{fl/fl}}$*Lyz2*-Cre (*Cd300a*$^{\text{fl/fl}}$, n = 6, 13, 11, 11, 10, 6, 6, 6, and 6; *Cd300a*$^{\text{fl/fl}}$*Lyz2*-Cre, n = 7, 17, 13, 12, 12, 12, 11, 11 and 11, respectively). (**G**) Sirius-red staining of the kidney 49 d after uIRI. Fibrosis in the tubulointerstitial area in *Cd300a*$^{\text{fl/fl}}$ (n = 6) and *Cd300a*$^{\text{fl/fl}}$*Lyz2*-Cre (n = 10) mice. The scale bars indicate 100 μm. Data are presented as means ± SEM, representative of 3 experiments (A) and pooled from 2 (C and E), 3 (B) and

4 (F and G) experiments. Statistical analyses were performed using two-way ANOVA (B, E) and unpaired Student's *t*-test (C, F and G). *, $P < 0.05$; **, $P < 0.01$; ***, $P < 0.001$.

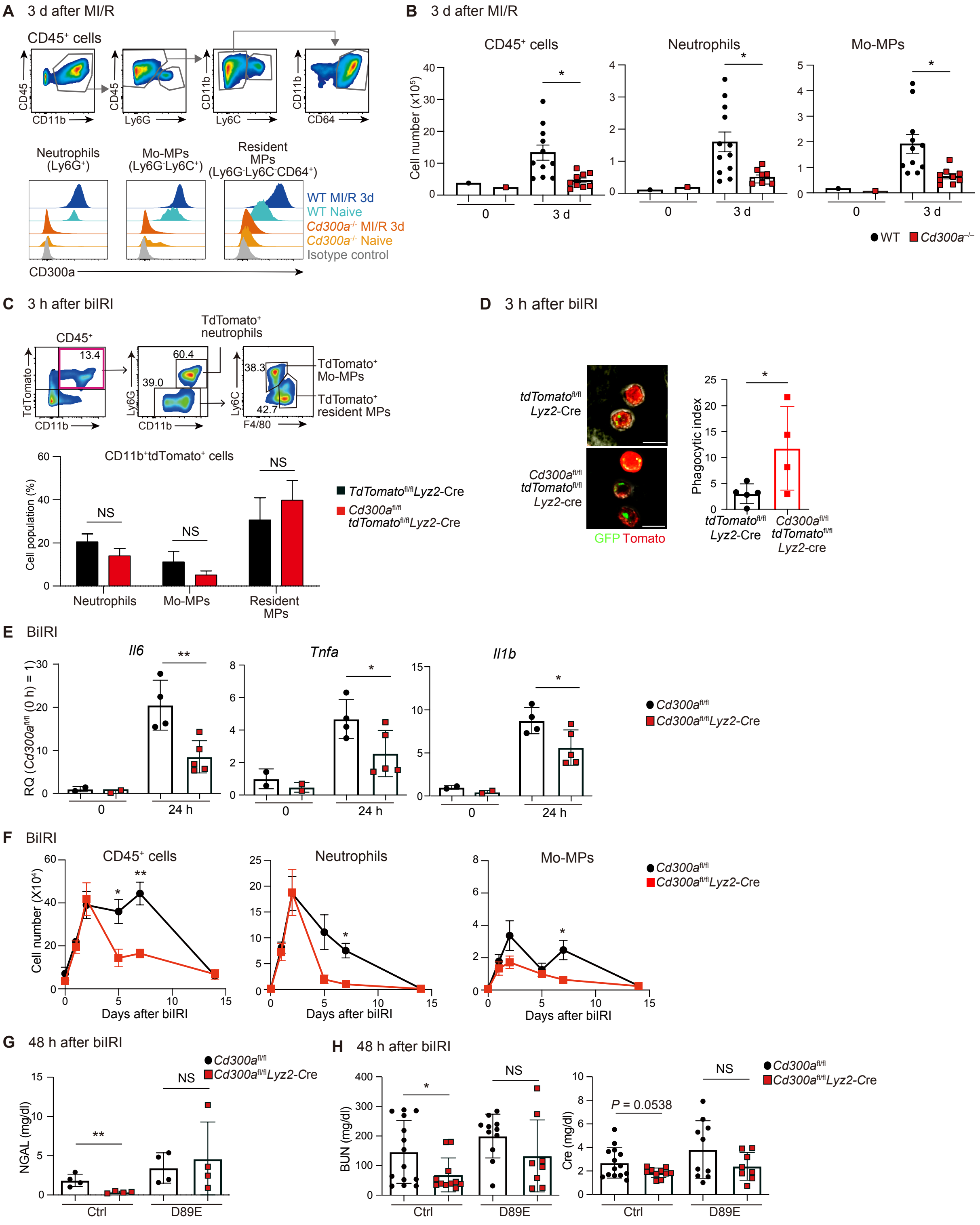

Fig. S3. CD300a expression on cardiac myeloid cells and enhanced efferocytosis by CD300a-deficient macrophages reduces tissue damages and fibrosis and preserves organ function after AKI.

(**A**) Flow cytometric analysis for CD300a expression on myeloid cells of neutrophils, monocyte-derived macrophages (Mo-MPs) and resident macrophages (resident MPs) in the cardiac tissue before (naive) or 3 days after MI/R. Representative of 3 experiments. (**B**) Flow cytometric analysis of immune cell populations (total CD45^{+} cells, CD11b^{+}Ly6G^{+} neutrophils and CD11b^{+} Ly6C^{+} Mo-MPs) in the cardiac tissue 3 days after MI/R in *Cd300a*$^{-/-}$ mice (n =

11) and WT mice (n = 12). (**C**) Gating strategy (top) and the percentage (bottom) of each myeloid cell subpopulations of neutrophils, Mo-MPs and resident MPs of $CD11b^+tdTomato^+$ cells in the kidney 3 h after biIRI. (**D**) Representative confocal laser scanning microscopy analysis of donor-derived $tdTomato^+CD11b^+$ myeloid cells engulfing host-derived GFP^+ dead cells in the kidney in the chimeric mice as shown in Figure 3D, 3 h after biIRI (left). Quantitative data of the percentage of GFP^+ dead cells-containing myeloid cells ($tdTomato^{fl/fl}$ *Lyz2*-Cre, n = 5; $Cd300a^{fl/fl}tdTomato^{fl/fl}$*Lyz2*-Cre, n = 4 in each group) (right). (**E**) Il6, Tnfa and Il1b expressions in $CD11b^+$ cells of kidneys before (0) and 24 h after biIRI in $Cd300a^{fl/fl}$ (n = 2 and 4, respectively) and $Cd300a^{fl/fl}$ Lyz2-Cre (n = 2 and 5, respectively) mice. (**F**) Cell numbers of $CD45^+$ cells, neutrophils and Mo-MPs in the kidneys of $Cd300a^{fl/fl}$ and $Cd300a^{fl/fl}$*Lyz2*-Cre mice before (0) and 1, 2, 5, 7, and 14 d (n = 2, 5, 6, 3, 5 and 5 in both genotypes, respectively) after biIRI. (**G** and **H**) Plasma levels of NGAL (G) and BUN and Cre (H) (Ctrl, n = 4; D89E, n = 4 for (G) and Ctrl, n = 14; D89E, n = 11; D89E, n = 8 for (H)) in control (Ctrl) or D89E-MFGE8 (D89E)-administered $Cd300a^{fl/fl}$ and $Cd300a^{fl/fl}$*Lyz2*-Cre mice 48 h after biIRI. Data are presented as means ± SEM and pooled of 2 (C and E), 3 (B and F) and 5 (G and H) experiments. Statistical analyses were performed using two-way ANOVA (B, C and E–H) and unpaired Student's *t*-test (D). *, $P < 0.05$; **, $P < 0.01$; NS, not significant.

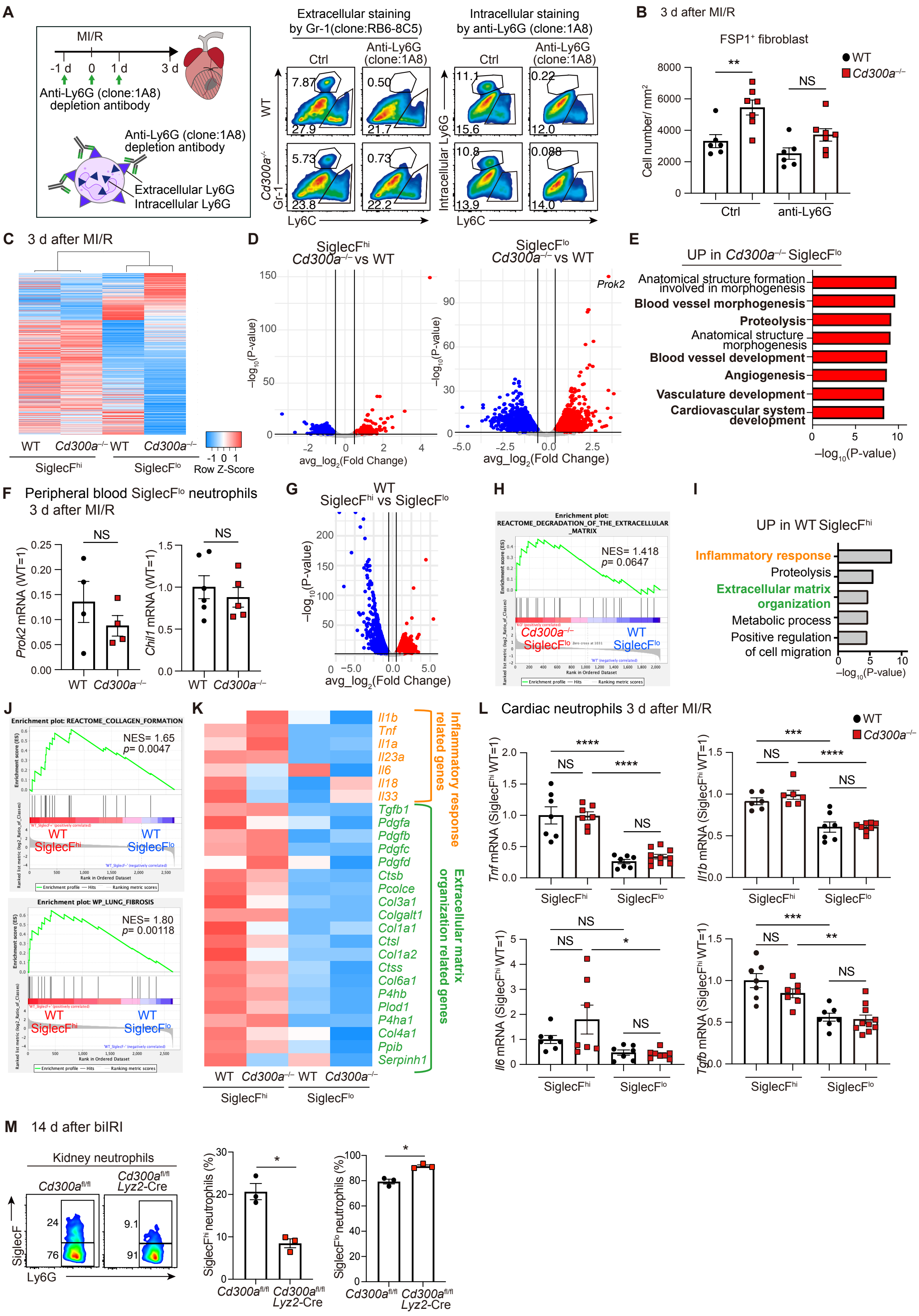

Fig. S4. No involvement of CD300a in pro-inflammatory SiglecF^{hi} neutrophils in cardiac tissue after MI/R.

(**A**) Schematic diagram of neutrophil depletion by i.v. injection of an anti-Ly6G mAb or isotype-control (Ctrl) on day-1, 0, and 1 post-MI/R (left). Flow cytometric analysis of neutrophil depletion (right). Since an anti-Ly6G mAb (clone 1A8) was used for the depletion of neutrophils, depletion of neutrophils were confirmed by detection of both cell surface Ly6G using different epitope binding Ly6G antibody (anti-Gr-1 antibody, clone RB6-8C5) and intracellular Ly6G using the same clone (1A8). (**B**) The cell number of $FSP1^{+}vimentin^{+}$ fibroblasts 3 d after MI/R in ctrl or anti-Ly6G mAb-administered $Cd300a^{-/-}$ mice (Ctrl, n = 7; Anti-Ly6G, n = 8) and WT mice (Ctrl, n = 7; Anti-Ly6G, n =7). (**C**) Heat map of RNA expressions of $SiglecF^{hi}$ and $SiglecF^{lo}$ neutrophils in cardiac tissue 3 d after MI/R from WT and $Cd300a^{-/-}$ mice (n = 8 in both genotypes), standardized by Z-score. (**D**) Volcano plots showing the amount of change in gene expression in $Cd300a^{-/-}$ compared to WT in $SiglecF^{hi}$ (left) and $SiglecF^{lo}$ (right) neutrophils. (**E**) Enrichment gene ontology terms (DAVID (v2022q4)) of the up-regulated genes in $Cd300a^{-/-}$ $SiglecF^{lo}$ neutrophils compared to WT $SiglecF^{lo}$ neutrophils ranked by their adjusted *P*-values. (**F**) *Prok2, Chil1* mRNA expressions in peripheral blood neutrophils from $Cd300a^{-/-}$ mice (n = 5) and WT mice (n = 6) 3 d after MI/R, quantified by qRT-PCR. (**G**) Volcano plots showing the amount of change in gene expression in WT $SiglecF^{hi}$ neutrophils compared to WT $SiglecF^{lo}$. (**H**) GSEA profiles showing a significant enrichment of gene sets associated with degradation of the extracellular matrix (Reactome) in $Cd300a^{-/-}$ $SiglecF^{lo}$ neutrophils compared to WT $SiglecF^{lo}$ neutrophils. (**I**) Enrichment Gene Ontology terms (DAVID (v2022q4)) of the up-regulated genes in WT $SiglecF^{hi}$ neutrophils compared to WT $SiglecF^{lo}$ neutrophils. GO terms were ranked by their adjusted *P*-values. (**J**) GSEA profiles showing a significant enrichment of gene sets associated with collagen formation (upper panel) and lung fibrosis (WikiPathways) (lower panel) in WT $SiglecF^{hi}$ neutrophils compared to WT $SiglecF^{lo}$ neutrophils. (**K**) Heat map of selected enriched genes encoding pro-inflammatory cytokines (orange characters) and pro-fibrotic factors (green characters). (**L**) mRNA expression of inflammatory cytokines (*Tnf, Il1b, Il6*) and pro-fibrotic factor (*Tgfb*) in $SiglecF^{hi}$ and $SiglecF^{lo}$ neutrophils migrated to cardiac tissue in $Cd300a^{-/-}$ mice (n = 10) and WT mice (n = 7) 3 d after MI/R, quantified by qRT-PCR. (**M**) Flow cytometric analysis of SiglecF expression on neutrophils in the kidney (left) and the percentage of $SiglecF^{hi}$ and $SiglecF^{lo}$ neutrophils (right) in the renal tissue in $Cd300a^{fl/fl}$ (n = 3) and $Cd300a^{fl/fl}$*Lyz2*-Cre (n = 3) mice 14 d after biIRI. Data are presented as means ± SEM and pooled of 3 (B), 2 (F) and 5 (L) experiments. Statistical analyses were performed using two-way ANOVA (B and L) and unpaired Student's *t*-test (F and M). *, $P < 0.05$; **, $P < 0.01$; ***, $P < 0.001$; ****, $P < 0.0001$; NS, not significant.

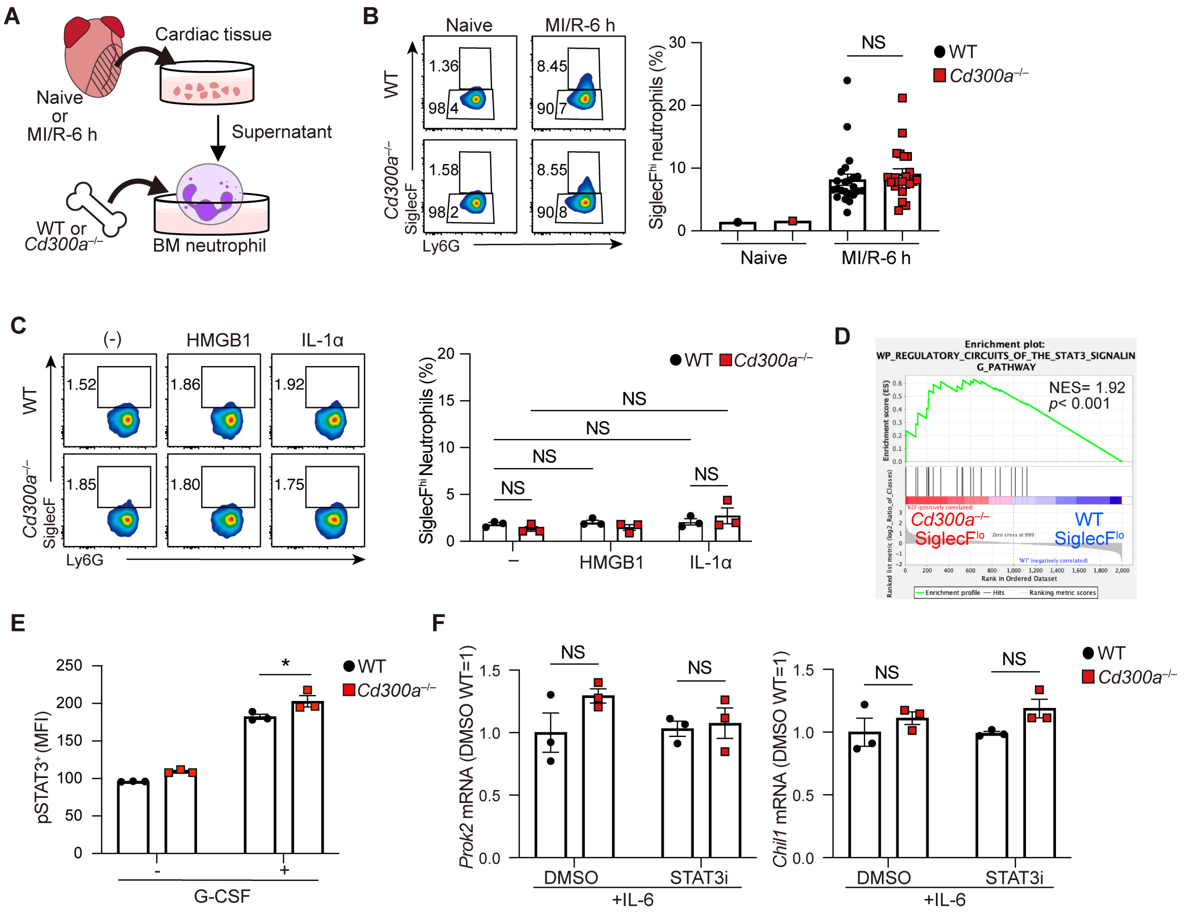

Fig. S5. SiglecF^{hi} neutrophils are induced by cardiac tissue environmental factors after MI/R.

(**A**–**C**) WT or *Cd300a*$^{-/-}$ neutrophils were stimulated for 2 d with the culture supernatant of the cardiac tissue before (naïve) or 6 h after MI/R (A and B) or with HMGB1 or IL-1α (C) and analyzed for SiglecF expression by flow cytometry (B, C). (**D**) Gene set enrichment analysis (GSEA) for STAT3 signaling pathways between WT and *Cd300a*$^{-/-}$ SiglecF^{lo} neutrophils. (**E**) Flow cytometric analysis of pSTAT3 levels in WT or *Cd300a*$^{-/-}$ BM neutrophils 1h after stimulation with or without G-CSF. (**F**) *Prok2* and *Chil1* mRNA expressions in WT or *Cd300a*$^{-/-}$BM neutrophils 12 h after stimulation with IL-6 (10 ng/ml) together with STAT3 inhibitor (STAT3i) or control vehicle (Vehicle). Data are presented as means ± SEM, pooled from 2 experiments (B) and representative of 2 (C and F) and 3 experiments (E). Statistical analyses were performed using one-way ANOVA (B, C, E and F).

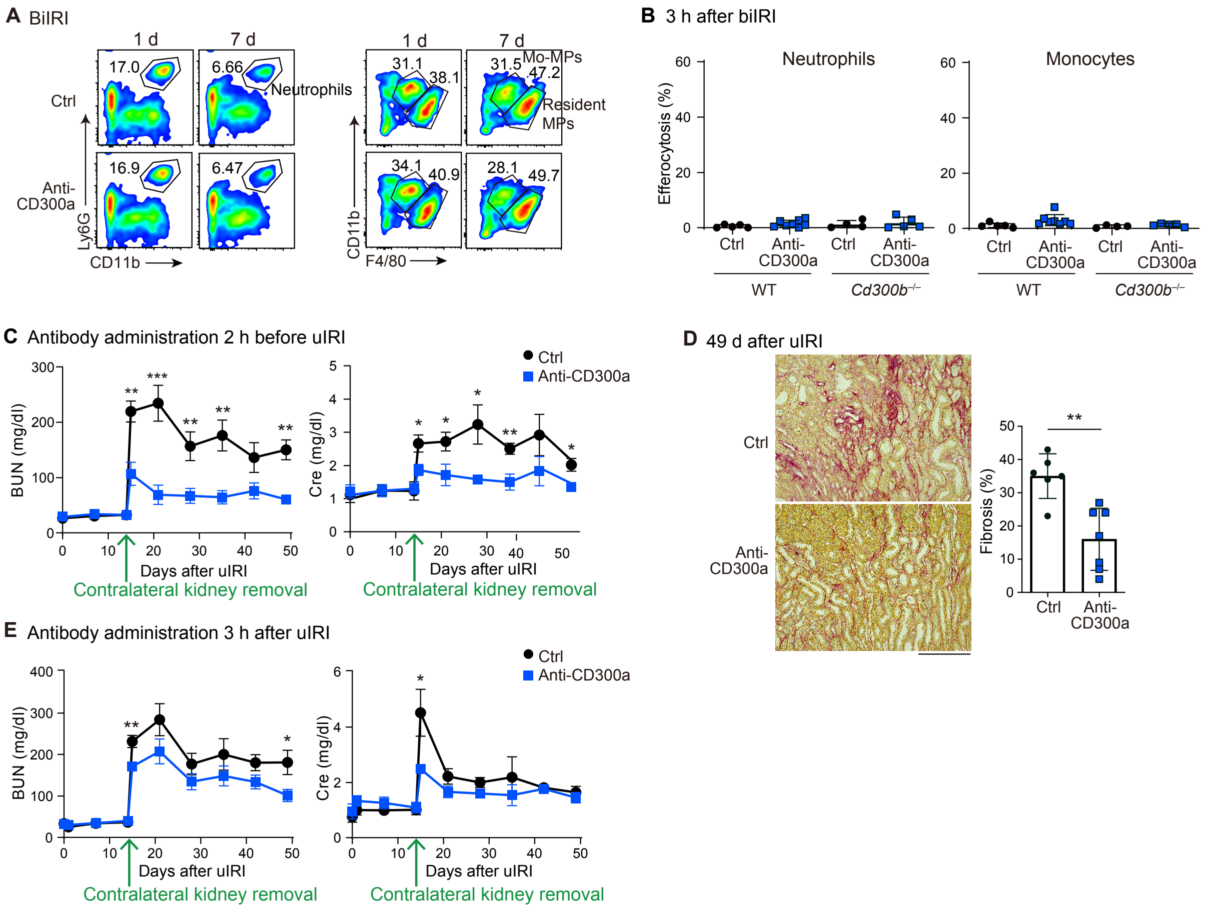

Fig. S6. A neutralizing mAb against CD300a ameliorates tissue injury and adverse remodeling after two-step uIRI.

(**A**) Flow cytometric analysis of CD45^{+} myeloid cell populations of neutrophils, monocyte-derived macrophages (Mo-MPs) and resident macrophages (resident MPs) in the renal tissue 1 and 7 days after biIRI that have been i.v. injected with anti-CD300a mAb or control antibody (Ctrl). Representative of 2 experiments. (**B**) R26GRR mice (GFP^{+} mice in Figure3D) received total body irradiation and then i.v. transferred with bone marrow (BM) cells from WT or *Cd300b*$^{-/-}$ mice to generate BM chimeric mice. BiIRI was subjected to these mice after four weeks with i.v. administration of an anti-CD300a mAb (WT; n = 8, *Cd300b*$^{-/-}$; n = 5) or Ctrl (WT; n = 5, *Cd300b*$^{-/-}$; n = 4) at 2 h before biIRI. Flow cytometry analysis of donor-derived neutrophils and Mo-MPs engulfing GFP^{+} dead cells in the kidney in the chimeric mice 3 h after biIRI. (**C**) The plasma levels of BUN and Cre before (0), 7, 14, 14, 21, 28, 35, 42 and 49 d after two-step uIRI in mice that had been i.v. injected with anti-CD300a mAb (n = 3, 8, 8, 8, 8, 7, 7, 7 and 7, respectively) or Ctrl (n = 3, 8, 8, 8, 7, 8, 4, 4 and 4, respectively) 2 h before two-step uIRI. (**D**) Representative Sirius-red staining (left) and fibrosis in the kidneys 49 d after two-step uIRI in mice that had been i.v. injected with anti-CD300a mAb (n = 6) or Ctrl (n = 6) 2 h before two-step uIRI. The scale bar indicates 100 µm. (**E**) The plasma levels of BUN and Cre before (0), 7, 14, 14, 21, 28, 35, 42 and 49 d after two-step uIRI in mice that had been i.v. injected with anti-CD300a mAb (n = 3, 8, 8, 8, 8, 7, 7, 7 and 7, respectively) or Ctrl (n = 3, 8, 8, 8, 7, 8, 4, 4 and 4, respectively) 3 h after two-step uIRI. Data are presented as means ± SEM and pooled of 2 (C and E), 3 (B and F) and 5 (G and H) experiments. Statistical analyses were performed using two-way ANOVA (B, C and E–H) and unpaired Student's *t*-test (D). *, $P < 0.05$; **, $P < 0.01$; ***, $P < 0.001$.

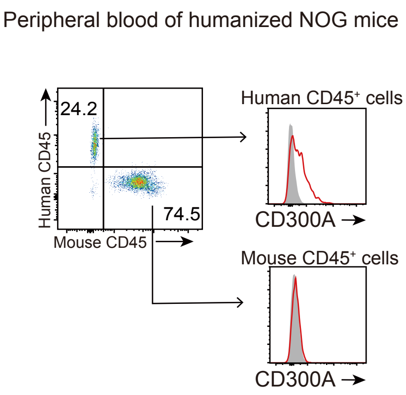

Fig. S7. CD300A expression in humanized mice.
Flow cytometric analysis of CD300A expression on human and mouse $CD45^+$ peripheral blood mononuclear cells in the humanized mice 48 h after biIRI.
